# Shining the spotlight on the neglected: new high-quality genome assemblies as a gateway to understanding the evolution of Trypanosomatidae

**DOI:** 10.1186/s12864-023-09591-z

**Published:** 2023-08-21

**Authors:** Amanda T. S. Albanaz, Mark Carrington, Alexander O. Frolov, Anna I. Ganyukova, Evgeny S. Gerasimov, Alexei Y. Kostygov, Julius Lukeš, Marina N. Malysheva, Jan Votýpka, Alexandra Zakharova, Kristína Záhonová, Sara L. Zimmer, Vyacheslav Yurchenko, Anzhelika Butenko

**Affiliations:** 1 Life Science Research Centre, Faculty of Science, University of Ostrava, 710 00 Ostrava, Czech Republic; 2 Department of Biochemistry, University of Cambridge, Tennis Court Road, Cambridge, CB2 1QW UK; 3 Zoological Institute of the Russian Academy of Sciences, 199034 St. Petersburg, Russia; 4 Faculty of Biology, M. V. Lomonosov Moscow State University, 119991 Moscow, Russia; 5 Martsinovsky Institute of Medical Parasitology, Sechenov University, 119435 Moscow, Russia; 6 Institute of Parasitology, Czech Academy of Sciences, 370 05 České Budějovice, Czech Republic; 7 Faculty of Sciences, University of South Bohemia, 370 05 České Budějovice, Czech Republic; 8 Department of Parasitology, Faculty of Science, Charles University, 128 44 Prague, Czech Republic; 9 Department of Parasitology, Faculty of Science, Charles University, BIOCEV, 252 50 Vestec Czech Republic; 10 Division of Infectious Diseases, Department of Medicine, University of Alberta, Edmonton, T6G 2G3 Canada; 11 Duluth Campus, University of Minnesota Medical School, Duluth, MN 55812 USA

**Keywords:** Trypanosomatids, Genome assembly, Whole-genome sequencing, Monoxenous, Dixenous, Parasite, Protist

## Abstract

**Background:**

Protists of the family Trypanosomatidae (phylum Euglenozoa) have gained notoriety as parasites affecting humans, domestic animals, and agricultural plants. However, the true extent of the group's diversity spreads far beyond the medically and veterinary relevant species. We address several knowledge gaps in trypanosomatid research by undertaking sequencing, assembly, and analysis of genomes from previously overlooked representatives of this protistan group.

**Results:**

We assembled genomes for twenty-one trypanosomatid species, with a primary focus on insect parasites and *Trypanosoma* spp. parasitizing non-human hosts. The assemblies exhibit sizes consistent with previously sequenced trypanosomatid genomes, ranging from approximately 18 Mb for *Obscuromonas modryi* to 35 Mb for *Crithidia brevicula* and *Zelonia costaricensis*. Despite being the smallest, the genome of *O. modryi* has the highest content of repetitive elements, contributing nearly half of its total size. Conversely, the highest proportion of unique DNA is found in the genomes of *Wallacemonas* spp., with repeats accounting for less than 8% of the assembly length. The majority of examined species exhibit varying degrees of aneuploidy, with trisomy being the most frequently observed condition after disomy.

**Conclusions:**

The genome of *Obscuromonas modryi* represents a very unusual, if not unique, example of evolution driven by two antidromous forces: i) increasing dependence on the host leading to genomic shrinkage and ii) expansion of repeats causing genome enlargement. The observed variation in somy within and between trypanosomatid genera suggests that these flagellates are largely predisposed to aneuploidy and, apparently, exploit it to gain a fitness advantage. High heterogeneity in the genome size, repeat content, and variation in chromosome copy numbers in the newly-sequenced species highlight the remarkable genome plasticity exhibited by trypanosomatid flagellates. These new genome assemblies are a robust foundation for future research on the genetic basis of life cycle changes and adaptation to different hosts in the family Trypanosomatidae.

**Supplementary Information:**

The online version contains supplementary material available at 10.1186/s12864-023-09591-z.

## Background

The kinetoplastid family Trypanosomatidae comprises parasitic flagellates that infect a diverse range of hosts, encompassing vertebrates, arthropods, leeches, plants, and even ciliated protists [1, 2]. For decades, trypanosomatid research was focused on the species causing diseases in humans, domestic animals, and agricultural plants, effectively neglecting the rest of the group. This resulted in significant knowledge gaps, with the first issue being a near absence of genomic data for monoxenous (one-host) members of the family, which predominantly infect insects [3]. Yet, these data are indispensable for understanding the evolutionary transitions from monoxeny to dixeny, which occurred at least three times independently in the evolution of Trypanosomatidae, in the vertebrate-parasitic *Leishmania*, *Trypanosoma*, and plant-infecting *Phytomonas* [4]. In addition, monoxenous trypanosomatids are characterized by an impressive adaptability to various insect hosts worldwide, even though the genetic background of such plasticity is not well understood. The situation has improved in recent years with reports of reference genome sequences for several insect parasites, including representatives of the genera *Blastocrithidia*, *Crithidia*, *Herpetomonas*, *Leptomonas*, *Novymonas*, *Paratrypanosoma*, and *Vickermania* [5–12]. In-depth analysis of these genomes has proven to be instrumental for shedding light on various aspects of trypanosomatid, and, more generally, eukaryotic biology. For instance, it led to the identification of novel virulence factors in human parasites of the genus *Leishmania* [7, 13], understanding the metabolic cooperation between trypanosomatids and their bacterial endosymbionts [11, 14, 15], as well as the discovery of novel mechanisms enabling stop-to-sense codon reassignment [12]. Despite recent progress in mitigating the bias towards practically relevant pathogens, the knowledge about several monoxenous genera is still restricted to formal taxonomic descriptions with sequencing data confined to some common phylogenetic markers, such as genes for 18S rRNA, glycosomal glyceraldehyde phosphate dehydrogenase, and spliced leader RNA [16, 17]. Consequently, the exact phylogenetic position of some trypanosomatid genera, e.g., *Sergeia*, *Wallacemonas*, and *Jaenimonas* [18–20] remains to be established using whole-genome data.

The second significant gap in trypanosomatid research pertains to the paucity of information on the diversity and biology of dixenous trypanosomatids that do not infect humans. The bulk of *Trypanosoma* research is focused on salivarian species (*Trypanosoma brucei*, *T. congolense*, and *T. vivax*), as well as the *T. cruzi* complex, causing severe diseases in humans and domestic animals [21–23]. Yet, the genus *Trypanosoma* is very species-rich, with its members isolated from a variety of sources, including amphibians, birds, fishes, mammals, and reptiles [1].

Lastly, obtaining genome sequences for the closest relatives of medically-, veterinary-, and agriculturally-significant species can provide insight into host switches and life cycle changes in trypanosomatids. Despite the fact that several genomes of these flagellates are currently available, they are not always ideally suitable for comparative analysis. For instance, the closest known relative of the dixenous genus *Leishmania*, is the monoxenous trypanosomatid *Novymonas esmeraldas*, whose gene content and metabolism appear to be affected by the presence of an endosymbiotic bacterium [11].

**Fig. 1:**
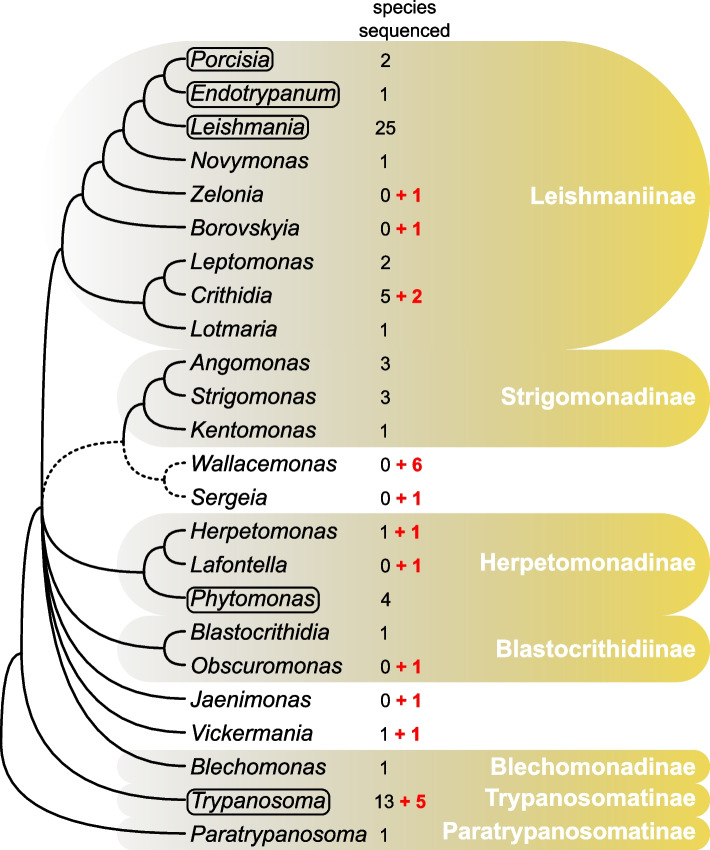
A cladogram depicting the phylogenetic relationships among trypanosomatids based on the available literature. The number of species within each genus with publicly available genomes is indicated, with the numbers in red representing the genomes sequenced in this study. The dixenous genera are displayed in boxes. Subfamilies are highlighted with a yellow background and their names are shown on the right. Not resolved relationships are indicated by dashed lines

In this study, we aim to address the gaps mentioned above by presenting genome assemblies for twenty-one species of the family Trypanosomatidae, including sixteen monoxenous representatives (Fig. 1). We report the genomes of monoxenous *Zelonia costaricensis* and *Borovskyia barvae*, close relatives of dixenous *Leishmania* [24, 25], as well as that of *Obscuromonas modryi*, a member of the genus sister to *Blastocrithidia* spp., which have all three stop codons reassigned as sense [12, 26, 27]. Additionally, we assembled the genomes of five non-human infective *Trypanosoma* spp. of four subgenera (*Haematomonas*, *Squamatrypanum*, *Trypanomorpha* and *Trypanosoma*). These data will provide insight into the phylogenetic relationships between these dixenous parasites and highlight genetic changes associated with host switches. We also report several genomes for representatives of the monoxenous genera *Crithidia* and *Wallacemonas*, including *C. thermophila*, a species capable of withstanding elevated temperatures [28] and *Wallacemonas* sp. TrypX, which was isolated from a rodent host [29]. The genomes of two representatives of the genus *Herpetomonas*, the closest known relatives of the plant-infecting *Phytomonas* spp. can shed light on the origin of this peculiar dixenous genus. For *Sergeia podlipaevi* and *Wallacemonas* spp., the presented genomes will be instrumental in ascertaining their phylogenetic position, while in the case of *Jaenimonas drosophilae* it is necessary for the scrutiny of host-parasite interactions in the *Jaenimonas*-*Drosophila* experimental model [20].

## Results

### *Genome assemblies for monoxenous trypanosomatids and* Trypanosoma* spp.*

The family Trypanosomatidae comprises over twenty recognized genera [1]. However, the majority of sequenced genomes belong to just two of them encompassing medically relevant species of the genera *Leishmania* and *Trypanosoma* [30]. We have obtained genome assemblies for representatives of ten trypanosomatid genera, including five (*Borovskyia*, *Jaenimonas*, *Sergeia*, *Wallacemonas*, and *Zelonia*), for which no such data were previously available (Table 1). For most species, the isolates used in this work have been obtained from insect hosts: such as Hemiptera (*B. barvae*, *C. thermophila*, *H. samuelpessoai*, *O. modryi*, *W. rigidus*, *W. collosoma*, *Wallacemonas* sp. Wsd and *Z. costaricensis*) and Diptera (*C. brevicula*, *J. drosophilae*, *S. podlipaevi*, *V. spadyakhi*, *Wallacemonas* sp. 195SL and MBr04). However, *Wallacemonas* sp. strain TrypX was isolated from a rodent [29], and therefore, might represent another example of a monoxenous trypanosomatid adapted to survival at elevated temperatures of a mammal body in addition to *Leptomonas seymouri* and *Crithidia thermophila* [28, 31]. It remains to be elucidated what is the molecular basis of this adaptation. We also sequenced genomes of several trypanosomes from non-human hosts, such as lizards, a toad, a ray, and a bird (Table 1).

**Table 1:** Sources of trypanosomatid cultures

#	Species	Strain	Host	Collection year and location
1	*Borovskyia barvae*	21EC	*Collaria oleosa* (Hemiptera)	2003, Costa Rica: Heredia
2	*Crithidia brevicula*	S14	*Heleomyza* sp. (Diptera)	2018, Russia: Sob’ village, Yamalo-Nenets Autonomous Area (67°06 N, 65°61 E)
3	*Crithidia thermophila*	CT-IOC 054	*Zelus leucogrammus* (Hemiptera)	1997, Brazil: Goiânia
4	*Herpetomonas samuelpessoai*	ATCC 30252	*Zelus leucogrammus* (Hemiptera)	1970, Brazil
5	*Herpetomonas tarakana*	OSR18	*Ectobius lapponicus* (Blattodea)	2014, Czech Republic: Šilheřovice, Černý les Nature Reserve
6	*Jaenimonas drosophilae*	Fi-01.02	*Drosophila falleni* (Diptera)	2006, USA: Vicinity of West Hartford, Connecticut (41°46′04″N, 72°45′14″W)
7	*Obscuromonas modryi*	Fi-14	Alydinae gen sp. (Hemiptera)	2013, Philippines: Bontoc (17°05′58"N, 120°59′22"E)
8	*Sergeia podlipaevi*	CER4	*Culicoides festivipennis* (Diptera)	2000, Czech Republic: Milovicky forest (48°49′39"N, 16°42′38"E)
9	*Trypanosoma avium* (*Trypanomorpha*)	A1412	*Corvus frugilegus* (Passeriformes)	1979, Czech Republic: Prague (49°59′12"N, 14°35′46"E)
10	*Trypanosoma boissoni* (*Haematomonas*)	ITMAP 2211	*Zanobatus atlanticus* (Myliobatiformes)	1969, Senegal: Green Cape, Dakar
11	*Trypanosoma mega* (*Trypanosoma*)	ATCC 30038	*Bufo regularis* (Anura)	Unknown, Africa
12	*Trypanosoma platydactyli* (*Squamatrypanum*)	RI-340	*Tarentola mauritanica* (Squamata)	2021, Italy: Bari (41˚03′04"N, 16˚53′39"E)
13	*Trypanosoma scelopori* (*Squamatrypanum*)	H3-2	*Sceloporus jarrovi* (Squamata)	1995, USA: Southfork Canyon, Chiricahua Mountains, Cochise County, Arizona
14	*Vickermania spadyakhi*	S13	*Nemopoda nitidula* (Diptera)	2020, Russia: Sob’ railway station, Yamalo-Nenets Autonomous Area (67° 06′ N, 65° 71′ E)
15	*Wallacemonas collosoma*	ATCC30261	*Limnoporus dissortis* (Hemiptera)	1960, USA: Minnesota, Minneapolis
16	*Wallacemonas rigidus*	Sld	*Saldula pallipes* (Hemiptera)	2001, Russia: Cape Kartesh, Chupa bay, White Sea coast, Karelia
17	*Wallacemonas* sp.	MBr04	*Cyrtoneuropsis conspersa* (Diptera)	2015, Brazil, Angra dos Reis (23° 0′28.74"S, 44°18′46.76"W)
18	*Wallacemonas* sp.	TrypX	*Rattus norvegicus* (Rodentia)	1983, Egypt: Alexandria
19	*Wallacemonas* sp.	Wsd	*Salda littoralis* (Hemiptera)	2001, Russia: Cape Kartesh, Chupa bay, White Sea coast, Karelia
20	*Wallacemonas* sp.	195SL	*Sarcophaga carnaria* (Diptera)	2018, Russia: Karelia, near Lakhdenpokhya town
21	*Zelonia costaricensis*	15EC	*Ricolla simillima* (Hemiptera)	2003, Costa Rica

**Fig. 2:**
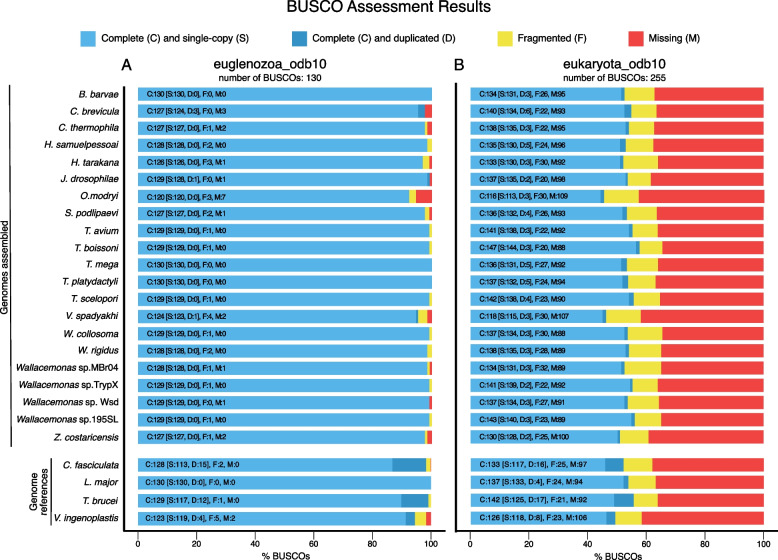
Genome assembly completeness. Presence of Benchmarking Universal Single-Copy Orthologs (BUSCOs) from two reference databases: specific Euglenozoa_odb10 (panel **A**) and more general Eukaryota_odb10 (panel **B**) is shown

**Fig. 3:**
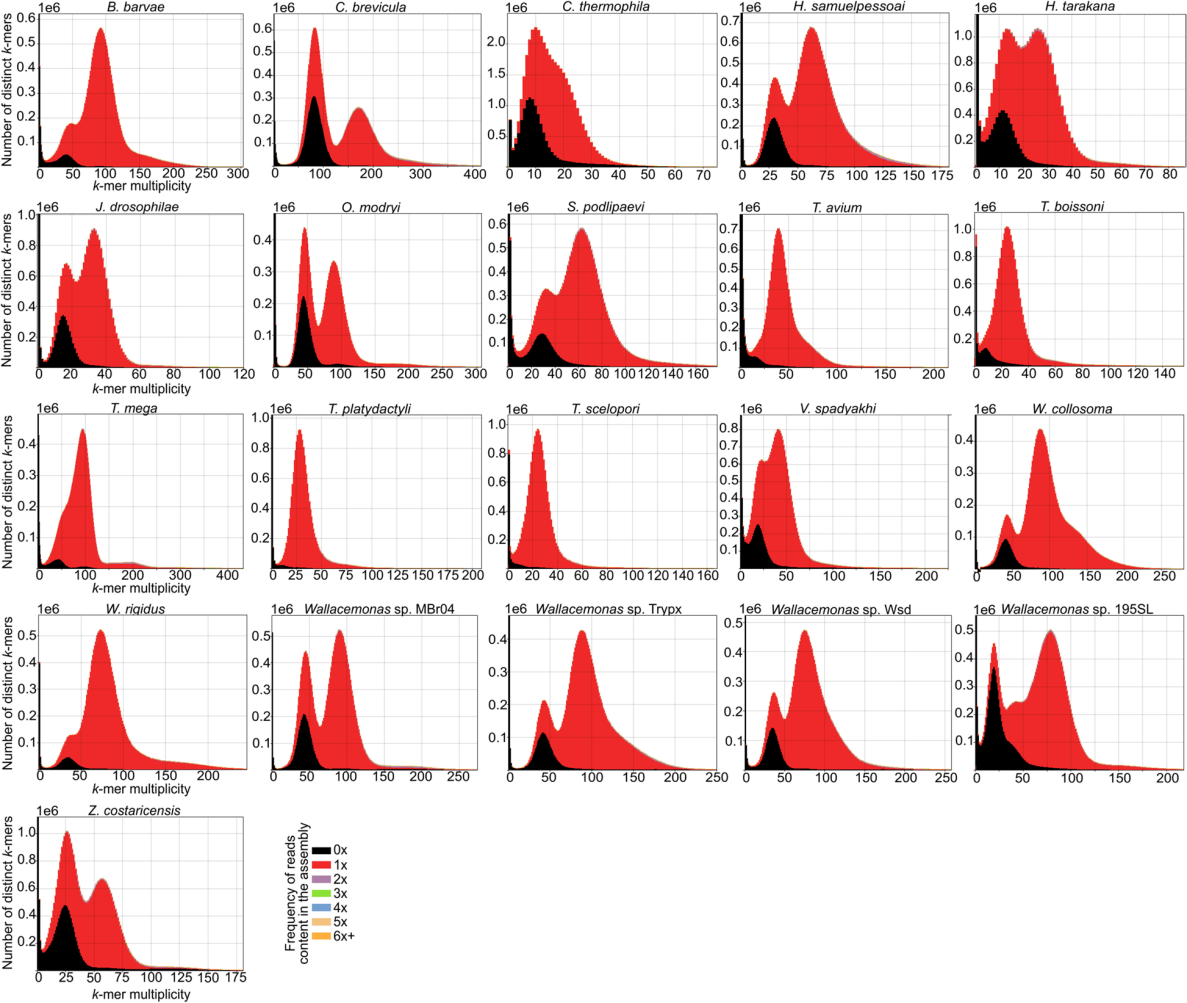
Spectra copy number plots for the genome assemblies. The multiplicity and number of distinct *k*-mers are plotted on the X and Y axes, respectively. The colors indicate the number of times each read is found in the genome assembly

These assemblies are based solely on Illumina data, although two different strategies were employed to ensure optimal results (see Materials and Methods for details). The shortest assembly is that of *O. modryi* (18.2 Mb), and the longest ones are those of *C. brevicula* (35.4 Mb) and *Z. costaricensis* (35.3 Mb) (Table 2). In each case, a significant proportion of the assembly is transcribed, ranging from 71 to almost 100% for *B. barvae* and *T. scelopori*, respectively (Additional file 1). Regardless of the size, almost all assemblies have BUSCO scores comparable to those for the reference trypanosomatid genomes (Fig. 2; Additional file 1). Only the assemblies of *O. modryi* and *C. brevicula* have slightly higher percentages of missing BUSCOs than other species and the reference genomes: 5.4% and 2.3%, respectively, when using Euglenozoa database (Fig. 2; Additional file 1). The high level of completeness of the assemblies and a high level of coherence between the genome assemblies and the reads used to produce them are supported by the results of the *k*-mer analysis (Fig. 3). The proportion of reads included into the final assembly ranges from 96.74% to 99.22% for *H. takarana* and *W. rigidus*, respectively (Fig. 3; Additional file 1). The percentage of genomic reads mapping back to the assembly ranges from ~ 86% to 100% for *C. thermophila* and *H. samuelpessoai*, respectively (Table 2; Additional file 1). The assembly error content is minimal as estimated based on the number of homozygous SNPs per 100 kb of genomic sequence ranging from 0.32 for *Wallacemonas* sp. TrypX to 1.49 for *V. spadyakhi* (Additional file 1).

**Table 2:** Genome assembly statistics

#	Species	Total assembly length, Mb^a^	N50, kb	% of missing BUSCOs^b^	% of genomic reads mapping back to the assembly	N's per 100 kb
1	*B. barvae*	32.9	181.4	0	99.8	24.5
2	*C. brevicula*	35.4	118.6	2.3	94.9	19.4
3	*C. thermophila*	30.0	47.7	1.5	85.6	6.7
4	*H. samuelpessoai*	32.2	128.4	0	100.0	11.3
5	*H. tarakana*	26.7	48.8	0.8	98.4	42.8
6	*J. drosophilae*	21.4	60.6	0.7	98.8	16.3
7	*O. modryi*	18.2	68.8	5.4	94.9	22.1
8	*S. podlipaevi*	26.9	45.7	0.8	87.2	14.4
9	*T. avium*	22.1	89.6	0	98.4	77.4
10	*T. boissoni*	22.2	84.0	0	98.6	13.7
11	*T. mega*	27.4	93.9	0	99.6	7.2
12	*T. platydactyli*	20.5	85.2	0	99.2	19.9
13	*T. scelopori*	20.3	59.1	0	99.0	62.0
14	*V. spadyakhi*	29.3	56.6	1.5	91.8	9.4
15	*W. collosoma*	25.7	167.3	0	98.7	4.9
16	*W. rigidus*	25.9	231.6	0	99.4	6.4
17	*Wallacemonas* sp. MBr04	27.7	114.4	0	95.3	6.8
18	*Wallacemonas* sp. TrypX	25.4	139.4	0	95.6	9.8
19	*Wallacemonas* sp. Wsd	26.4	103.1	0.8	94.5	14.9
20	*Wallacemonas* sp. 195SL	27.3	64.5	0	98.6	20.7
21	*Z. costaricensis*	35.3	41.4	1.5	89.5	22.6

^a^All statistics are based on scaffolds ≥ 500 bp

^b^Euglenozoa_odb10 used as a database

Importantly, although all the cultures except that of *B. barvae* were axenic, as a precautionary measure, we screened all genome assemblies for contamination. A few potential contaminating sequences were filtered out from the final assemblies of *B. barvae*, *O*. *modryi*, and all trypanosomes (Additional file 2). In the latter case, a contamination by vertebrate DNA can be explained by cultivation on blood agar (see Materials and Methods).

#### Analysis of repetitive elements

It is widely recognized that trypanosomatids possess a highly similar repertoire of protein-coding genes, and demonstrate a striking conservation of gene order, known as synteny [32]. An intriguing characteristic that is both remarkably divergent and significantly understudied across trypanosomatid genomes is the extent to which repetitive elements contribute to genome size [33]. To investigate whether there is a discernible pattern across trypanosomatids regarding the prevalence of repetitive elements, we examined this characteristic in all assembled genomes.

**Fig. 4:**
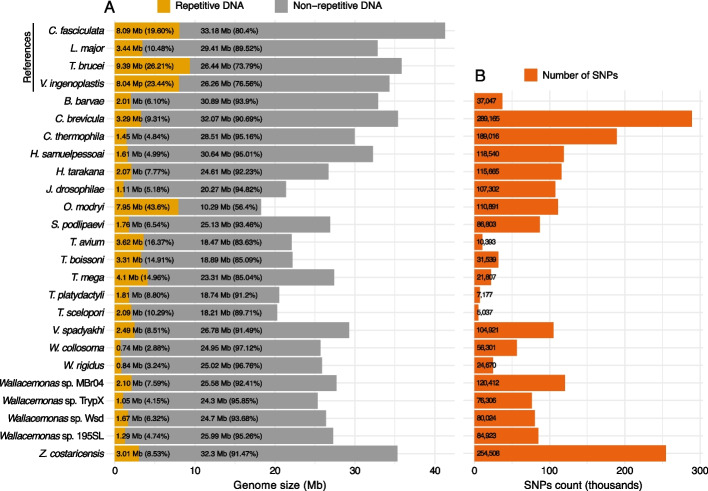
Content of repetitive DNA and single nucleotide polymorphisms in the genome assemblies. **A** Bar plot showing proportions of repetitive (dark yellow) and unique DNA (gray). The assembly size in megabases is also indicated for each species on X axis. **B** Total number of SNPs identified for each genome assembly. Bar charts were produced with the R v. 4.3 package ggplot2

Among analyzed assemblies, repetitive DNA is the least abundant in those of *W. collosoma* and *W. rigidus*, with repeats representing 2.88% and 3.24% of the total length, respectively (Fig. 4A; Additional file 3). In general, genome assemblies of *Wallacemonas* spp. are characterized by low repetitive content, with the maximal value of 7.59% documented in the MBr04 strain. The highest proportion of this content is represented by simple and unclassified repeats. In contrast, despite the smallest assembly size, *O. modryi* contains nearly 44% repetitive content, of which (among categorized repetitive content) retroelements (mainly LTRs and LINEs) are the most frequent (~ 12%).

The repetitive content varies greatly among *Crithidia* spp. The reference assembly of *C. fasciculata* contains almost 20% of the repeats, followed by *C. brevicula* with ~ 9% of them, and, finally, *C. thermophila* demonstrating only ~ 5%. *Herpetomonas* genomes also differ: *H. tarakana* possesses a shorter assembly and a higher repetitive content compared to *H. samuelpessoai*, with most differences originating in proportions of simple repeats (Fig. 4A; Additional file 3)*.* The genomes of *Trypanosoma* spp. assembled in this study show medium repeat content, ranging from 8.80% to 16.37% in *T. platydactyli* and *T. avium*, respectively (Fig. 4A; Additional file 3). Repetitive elements in the genome assemblies of *T. avium* and *T. scelopori* consist of mostly simple repeats, while in *T. platydactyli* retroelements (LTRs and LINEs) are predominant (Fig. 4A; Additional file 3).

The analysis of repeat content of the ten longest scaffolds for each species revealed a relatively even distribution of repeats (Additional file 4). The majority of the repeats identified on both the ten longest scaffolds and the entire assembly are transcribed (Additional files 3 and 4. The proportion of transcribed repeats varies, ranging from approximately 73% in *Wallacemonas* sp. 195SL to nearly 100% in *T. scelopori* (Additional file 3).

#### Single nucleotide polymorphisms and chromosome copy number variation

Variations in ploidy and heterozygosity are recognized as significant factors contributing to genome plasticity across various eukaryotes, including such distantly related organisms as trypanosomatids and fungi [34, 35]. Yet, the extent to which ploidy and heterozygosity drive genetic diversity in Trypanosomatidae is relatively well understood only for human pathogens [34, 36, 37]. Thus, we opted to investigate these features in the species sequenced in this study.

Variant calling allowed estimation of intragenomic variation across the assemblies produced here. The highest total SNP numbers, 289,423 and 254,508, were documented in the monoxenous representatives of the subfamily Leishmaniinae, *C. brevicula* and *Z. costaricensis*, respectively (Fig. 4B; Additional file 1). Conversely, all genomes of dixenous trypanosomatids sequenced herein demonstrated the lowest SNP content, with the smallest numbers being 5,037 and 7,177 for *T. scelopori* and *T. platydactyli*, respectively (Fig. 4B; Additional file 1). This correlates with the presence of only a single peak corresponding to homozygous content on the spectra copy number plots for the dixenous trypanosomatids, with the heterozygous peak being either entirely absent or barely visible (Fig. 3).

**Fig. 5:**
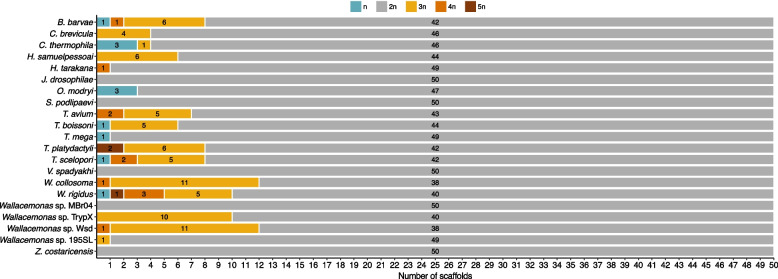
Stacked barplots showing somy estimates for 50 longest scaffolds for each trypanosomatid species. Numerical values in the plot indicate the number of scaffolds with each somy level. Somy estimation was performed based on the ratio of median-of-means coverage for 1 kb windows to the median genome coverage. Somy values are color coded: blue – monosomy, gray – disomy, dark yellow – trisomy, orange – tetrasomy, brown – pentasomy

We conducted coverage-based estimation of somy for the 50 longest scaffolds (used as chromosome proxies) for each species. Our analysis assumed that the median genome coverage reflects a disomic state. Only five out of twenty-one species with the assembled genomes appear to be diploid: *Z. costaricensis*, *J. drosophilae*, *V. spadyakhi*, *S*. *podlipaevi*, and *Wallacemonas* sp. MBr04 (Fig. 5; Additional files 5 and 6). Other genomes exhibit variable levels of aneuploidy, with the second most frequent state (after disomy) being trisomy. Aneuploidy is especially pronounced in *B. barvae* as well as in most *Trypanosoma* and *Wallacemonas* spp., where up to 25% of scaffolds exhibit notably altered coverage, in some cases consistent with tetra- and, even, pentasomy (Fig. 5). Importantly, in contrast to the aneuploidy in other species, the genomes of *O. modryi* and *T. mega* do not possess supernumerary chromosomes and their assemblies feature three and one scaffolds with reduced somy levels, respectively (Fig. 5; Additional files 5 and 6). In order to check if there is a correlation between somy levels and repeat content, we conducted a comparison between the proportion of repeats identified in disomic scaffolds and scaffolds with other somy levels (Additional file 5). However, we did not observe any statistically significant differences between the two groups (*p*-value < 0.01).

An independent approach for ploidy estimation, based on the analysis of heterozygous *k*-mer pairs, further supports our assumption that disomy is the prevailing state for each species (Additional file 7). This method considers all genomic information in trimmed sequencing reads, not just the largest scaffolds, and reveals the presence of *k*-mers indicative of aneuploidy even in species where diploidy was initially inferred using coverage analysis of the 50 largest scaffolds (Fig. 5, Additional files 5, 6 and 7. To fully comprehend whether the respective *k*-mers originate from chromosomes exhibiting states other than disomy or, for example, from segmental duplications, chromosome-level assemblies are necessary.

## Discussion

The assemblies obtained in this study cover a substantial number of monoxenous trypanosomatid genera with no previously available sequencing data, as well as several *Trypanosoma* spp. from non-human hosts (Fig. 1; Table 1). These data provide further evidence of the previously observed high genome size variability in trypanosomatids apparently reflecting distinct life strategies. The difference in size between the smallest (*O. modryi*) and largest (*C. brevicula* and *Z. costaricensis*) genome assemblies was almost twofold (Table 2). While we do not know much about the biology of the latter species, we suggest that one of the factors explaining the size difference between the remaining two can be the host specificity. Indeed, *O. modryi* (referred to as TU6/7 C before the formal description) is restricted to the heteropteran family Alydidae [26, 38]. In contrast, *C. brevicula* has a very wide host range and can successfully establish infections at least in phytophagous (Miridae) and predatory (Nabidae and Gerridae) true bugs, various flies (Antomyidae, Calliphoridae, Heleomyzidae, Muscidae, Sepsidae), as well as in mosquitoes (Culicidae) [19, 39–41]. Undoubtedly, the host range cannot be the only factor explaining these differences and a thorough analysis of the genomic composition should better clarify the underlying reasons.

Another highly variable characteristic of trypanosomatid genomes is the proportion of repetitive DNA [33]. We observe an approximately 15-fold span in estimates of repetitive DNA content among the assemblies obtained in this study: from 3% in *W. collosoma* to 44% in *O. modryi* (Fig. 4A; Additional file 3). The situation with the latter species is paradoxical: it has the highest proportion of repeats and the smallest genome size (Fig. 4A). In general, genome shrinkage can be stipulated by the growing dependence of an endosymbiont (including endoparasites) on the host or simplification of the lifestyle due to stable conditions (relevant also for free-living organisms) [42, 43]. Natural selection supports genome reduction in such conditions, because this allows faster genome replication [44]. Repetitive DNA is one of usual targets for genomic reduction, as demonstrated by studies elucidating the factors contributing to genome size variation in microsporidians, a large group of fungi-related unicellular eukaryotes infecting a wide range of hosts [45]. In *O. modryi*, on the contrary, we observe the expansion of repeats. This suggests the existence of an underlying strong evolutionary force competing with the one leading to genome shrinkage. Previously it has been argued that expansion of repetitive DNA and, in particular, simple repeats can enhance genome plasticity [46, 47] and here it also can be a case. A slightly higher proportion of missing BUSCOs compared to other assemblies indicates that the competing evolutionary forces driving *O. modryi* genome shrinkage are also quite prominent, which is evidenced by the loss of some protein-coding genes, which are typically highly conserved throughout Euglenozoa (Fig. 2). Repetitive DNA of trypanosomatids includes members of multigene families (e.g., variant surface glycoproteins, *trans*-sialidases, mucins), transposable elements, and satellite DNA repeats [33]. Before this work, *Trypanosoma* spp. were considered to have the highest proportions of repetitive DNA. For example, it comprises 51.3% of the *T. cruzi* genome and 20.7% (or ~ 26%, as estimated in our study) of that of *T. brucei* TREU927 [33]. Repeats in these species mainly consist of multigene families and retroelements [33]. Although our analyses did not include multigene families except for those of non-protein coding genes [48], we find that repetitive content of assemblies of *Trypanosoma* spp. of non-human hosts shows almost two-fold differences, from 9% in *T. platydactyli* to 16% in *T. avium* (Additional file 3). Furthermore, a substantial portion of the annotated content consists of simple repeats (up to 11% of the assembly in *T. avium*) and retroelements (up to 3% *T. platydactyli*) (Additional file 3). Although the proportion of repetitive content represented by multigene families has yet to be determined for the genomes sequenced in this study, our analyses suggest that the content of interspersed repeats and low complexity DNA sequences represents one of the factors responsible for intrageneric variations in trypanosomatid genome sizes. Most of the repeats identified in the analyzed assemblies are transcribed and appear to be evenly distributed along the longest scaffolds (Additional files 3 and 4). Of note, repeats, especially those exceeding the length of sequencing reads, can negatively affect assembly quality and lead to various artefacts in the subsequent analyses [49]. Therefore, the values we obtained for proportions of repetitive elements may be underestimated for our short-reads-based Illumina data, since some repeats might have been collapsed and/or not assembled.

Previous studies detected aneuploidy in *Leishmania* spp. [36, 50, 51], *T. cruzi* [52], *Leptomonas pyrrhocoris* [7], *Angomonas deanei* [53], *Endotrypanum* spp. and *Porcisia* spp. [54], as well as in *T. brucei brucei*, but not in the infective to humans *T. brucei gambiense* and *T. brucei rhodesiense* [55, 56]. However, it has never been systematically investigated for the whole family. Here we revealed aneuploidy in sixteen out of twenty-one genomes representing multiple lineages of Trypanosomatidae (Fig. 5) and, therefore, argue that this phenomenon is widespread among these flagellates. Trypanosomatids are predisposed to aneuploidy because, with only a few exceptions, they lack gene-specific transcriptional regulation for the majority of protein-coding genes [57]. It remains to be investigated further what mechanisms of tolerance to aneuploidy they have evolved, since the main problem to solve in this case is the meiotic segregation [58]. Whatever the solution can be, the advantage of chromosome copy number variation is a simple way to permanently change (increase or decrease, in case of poly- and monosomy, respectively) the expression of multiple genes, such as those coding, for example, for virulence factors, in an adaptation to changing environmental conditions or new hosts [59–61]. Nevertheless, the analysis of coverage using 50 largest scaffolds, led to the identification of several truly diploid species in our study (*Z. costaricensis*, *J. drosophilae*, *V. spadyakhi*, *S*. *podlipaevi*, and *Wallacemonas* sp. MBr04). Similarly to *T. brucei*, they may represent exceptions, rather than a rule within the family [62]. It remains to be elucidated why such species are diploid and how aneuploidy can affect sexual process in trypanosomatids. Of note, an independent approach to ploidy estimation, based on the analysis of heterozygous *k*-mer pairs, indicates that the putatively diploid species mentioned above show some indications of aneuploidy and/or other types of sequence duplication (Additional file 7).

Heterozygosity, the presence of two distinct alleles at a specific locus, is influenced by a number of factors including mode of reproduction [63] and ploidy [35]. In aneuploid fungal pathogens, loss of heterozygosity can stem from the chromosome gain with the subsequent loss of the heterozygous homolog [35]. Although we noticed that analyzed trypanosomes with lowest number of SNPs (*T. scleropori* and *T. platydactyli*) also have a relatively high aneuploidy level (with 8 out of 50 analyzed scaffolds showing somy levels different from disomy) (Fig. 5), we do not see a clear correlation between aneuploidy levels and loss of heterozygosity in trypanosomatids as observed in fungi [64]. This indicates that other factors in addition to ploidy variations define heterozygosity levels in these parasitic protists.

The assemblies generated in the course of this study do much to fill veritable canyons in the genetic and taxonomic record of trypanosomatids. However, important work remains to be done. The scaffolds assembled here are proxies for actual chromosomes. Assembly of full chromosomes will be desirable in the future to accurately infer genetic details such as chromosome copy number variations. Confirmation that most chromosomes in each genome are disomic will also validate our ploidy estimates for each species. However, until we or others generate such assemblies, the current work provides rich resources for many other types of biological comparisons within the richly varied trypanosomatids.

## Conclusions

In this work, we present genome assemblies for twenty-one trypanosomatid species, including overlooked monoxenous species and dixenous *Trypanosoma* spp. parasitizing non-human hosts. As judged from multiple standard metrics, our assemblies are highly contiguous and complete, making them valuable resource for various future analyses. We revealed relatively high intra- and intergeneric genome diversity in trypanosomatids in terms of size, repeat content, and ploidy. The new assemblies will be instrumental for establishing the molecular basis of tolerance to vertebrate host temperatures, elucidating impacts of life cycle changes and host switches on the genome, studying the origin of stop codon reassignment in Blastocrithidiinae, and many other phenomena that can now be approached using genomic data.

## Materials and methods

### DNA and RNA isolation

The sources of trypanosomatid cultures used in this work are specified in Table 1. All monoxenous species were cultivated at 23 °C in Schneider’s Drosophila medium (SDM) (Merck, St. Louis, USA) supplemented with 10% fetal bovine serum (Thermo Fisher Scientific, Waltham, USA), 100 μg/ml of streptomycin and 100 Units/ml of penicillin (Merck). *Trypanosoma* spp. were cultivated on biphasic blood agar overlaid with supplemented SDM.

Total genomic DNA was isolated from 10 ml of a culture either using DNeasy Blood & Tissue Kit (Qiagen, Hilden, Germany) according to the manufacturer’s instructions, or by the standard phenol–chloroform method. RNA isolation was performed using the RNeasy minikit (Qiagen) following the manufacturer’s protocol. Species identity was confirmed as in [65].

### Genome and transcriptome sequencing

DNA and RNA libraries were prepared and sequenced using Illumina instruments at Macrogen Europe (Amsterdam, Netherland), Institute of Applied Biotechnologies (Praha – Strašnice, Czech Republic), or Biomarker Technologies BMKGene (Münster, Germany) (Additional file 1). Depending on species, this yielded 14–128 million of 100 or 150 nt long paired-end genomic reads (Additional file 1). To facilitate future genome annotation process 16–93 million Illumina paired-end transcriptomic reads were produced for the same species.

### Genome assembly

Raw Illumina sequencing reads were adapter- and quality trimmed using Fastp v.0.20.1 [66] or BBDuk v.38.98 from BBMap package [67], and only paired-end reads with a minimum length of 75 and 50 nt were retained for further analysis in the case of genomic and transcriptomic data, respectively (Additional file 8, example command lines). Read quality and adapter content were assessed before and after the trimming with FastQC v.0.11.9 [68]. Genomic reads were subjected to a multiple sequence alignment-based error correction procedure using Karect [69]. The results of the error correction step were assessed with Karect ‘-align’ and ‘-eval’ on the preliminary assemblies. The read correction procedure resulted in the reduction of the area under the curve corresponding to the low-frequency *k*-mers estimated using KAT v.2.4.2 [70] with default settings.

Trimmed genomic reads were assembled de novo using two strategies and the best result was preserved. The Spades-Platanus strategy included using SPAdes v.3.13.0 [71] to assemble into contigs and Platanus v.1.2.4 [72] for scaffolding in two rounds intercalated with GapCloser v.1.12 module from SOAPde-novo2 for gap filling [73]. It showed better results for *J. drosophilae, H. tarakana, B. barvae, T. avium, T. scelopori, T. boissoni, T. mega, T. platydactyli, W. rigidus, W. collosoma, Wallacemonas* sp. 195SL, and *O. modryi.* The Platanus-solo strategy consisted in using Platanus for assembly and two-round scaffolding with gap filling in GapCloser. This strategy worked better for *C. thermophila, C. brevicula, Z. costaricensis, H. samuelpessoai, V. spadyakhi, S. podlipaevi, Wallacemonas* spp. MBr04, Wsd, and TrypX.

Overall, the selection of an assembly strategy for each species was carried out based on estimating the following assembly parameters: i) N_50_ as a measure of contiguity; ii) percentage of missing universal single-copy orthologs as a measure of completeness (BUSCO v.5 and Euglenozoa_odb10 and Eukaryota_odb10 as reference databases) [74]; iii) completeness estimated using *k*-mer analysis results produced using KAT v.2.4.2 [70]; iv) total gap length; v) percentage of homozygous SNPs as measure of accuracy; vi) size of the largest scaffold estimated using QUAST v.5.0.2 [75]; vii) percentage of sequencing reads mapping back to the assembly.

Trimmed transcriptomic reads were mapped to the assemblies using Bwa-mem2 [76], and the resulting alignments were sorted with SAMtools v.1.16.1 [77]. For estimating the transcribed portion of the genome, GTF files produced using Cufflinks v.2.2.1 [78] were analyzed with SeqKit v.0.16.1 [79].

### Assembly decontamination

The genome assemblies were checked for potential contamination with BlobTools v.1.1.1 [80]. The scaffolds satisfying the following criteria were discarded: 1) shorter than 500 nucleotides; 2) showing high-quality BLASTN hits (i.e., nucleotide sequence identity > 95% and query coverage > 85%) to non-euglenozoan sequences in NCBI nucleotide (nt) database (download date: 2022–05-08). Scaffolds with non-euglenozoan hits below the removal threshold were verified either using DIAMOND v.2.0.15 [81] in the sensitive mode or by BLASTX and kept in the final assembly if euglenozoan sequences were retrieved as best hits. The BLAST package v.2.13.0 was used for the homology searches mentioned above [82].

Different scaffold filtering criteria were applied to the genome assembly of *B. barvae*, since this species cannot be cultivated without accompanying yeast [83]. A preliminary assembly was produced, and the following scaffolds were removed: 1) with fungal sequences as best hits; 2) unannotated sequences demonstrating genomic read coverage below 63 × and grouping with fungal sequences according to the BlobTools analysis (Additional file 2). The genome was then re-assembled as described above using the reads mapping to the remaining scaffolds.

### Repeat analysis

The final genome assemblies were submitted to RepeatModeler v.2.0.3 [84] with the LTRStruct parameter for long terminal repeat (LTR) retroelements search. RepeatMasker v.4.1.2 [48] with sensitive slow search was used for repeats’ identification and soft masking using the database built with RepeatModeler. Statistical analysis of repeat content between disomic and other somy level scaffolds was performed using two-tailed *t*-test with a significance level of 0.01 for the species where at least two scaffolds demonstrated somy level distinct from 2n based on the coverage analysis.

For calculating the proportion of transcribed repeats, a GFF file with the repeat coordinates was used as an input for featureCounts v.2.0.1 [85] along with the BAM file containing transcriptomic reads mapped on the genome assembly. The read pairs mapping to the same scaffold and strand only once were counted. A repeat was considered transcribed if at least one read mapped to it.

J-Circos2 v.1.0 interface for Circos plot was used for visualization of repetitive content of the ten largest scaffolds for each species [86]. The GC skew was calculated for 1 kb non-overlapping windows using GCcalc [87], and the transcriptomic coverage track data was generated using bamCoverage v.3.5.2 implemented in deepTools2 software [88].

### Ploidy analysis

For each scaffold, mean read depths were calculated in successive non-overlapping 1 kb windows using Mosdepth v.0.3.3 with default settings [89] and then served to obtain a median-of-means (MOM) estimate. The median genome coverage was calculated based on those of the 50 largest scaffolds for each species. The ratio (R) between the scaffold’s MOM coverage and the median genome coverage was used to define somy: 0.25 ≥ R < 0.75 – monosomic; 0.75 ≥ R ≤ 1.25 – disomic; 1.25 > R ≤ 1.75 – trisomic, etc. The somy of each scaffold was inferred assuming that most of the scaffolds/chromosomes are in the disomic state. To provide a more detailed visualization of scaffold/chromosome copy number variation, the copy number was estimated for each of the 1 kb windows using the same strategy as above, but now dividing mean coverage within each 1 kb window by the genome median coverage (Additional file 6). The results were visualized using R packages ggplot2 v.3.4.2 and dplyr v.1.0.8 [49, 90].

In another approach to ploidy estimation, trimmed and corrected sequencing reads were used for *k*-mer analysis by KMC v.3.1.1 [91] with the subsequent ploidy inference using Smudgeplot v.0.2.5 [92].

### Variant calling

Trimmed and corrected genomic reads were mapped back to the assemblies using Bwa-mem2 [76], and the resulting alignments were sorted with SAMtools v.1.16.1 [77]. The mapping rates and median insert size were assessed with ‘stats’ from BamTools v.2.4.1 [93].

After mapping, genomic read duplicates were removed with MarkDuplicates, and the reads were locally realigned using IndelRealigner tools of GATK v.4.2 [94] with the default settings, except for REMOVE_DUPLICATES = true. Variant calling was performed using Platypus v.0.8.1 [95] with the default settings. Identified variants were extracted using GATK VariantsToTable.

### Supplementary Information

**Additional file 1. **Genome and transcriptome sequencing and assembly statistics. The genomes of *C. fasciculata* Cf-Cl, *L. major* Friedlin, *T. brucei brucei* TREU927 and *V. ingenoplastis* COLPROT021 are used for comparative purposes.

**Additional file 2. **Blobplots showing the state of genome assemblies before and after decontamination for the following species: *Borovskyia barvae* (panel A), *Obscuromonas modryi *(B), *Trypanosoma avium* (C), *Trypanosoma boissoni* (D), *Trypanosoma mega* (E), *Trypanosoma platydactyli* (F), and *Trypanosoma scelopori* (G).

**Additional file 3. **Repetitive content of genome assemblies. Overall statistics on the repetitive content and classification in all genomes assembled in this study and the references for comparison.

**Additional file 4. **J-circos plots showing the distribution of repeats along ten longest scaffolds for each trypanosomatid species. The tracks are the following (from inside out): repeat distribution, GC skew, transcriptomic read mapping, scaffold borders and IDs. Total length of the ten largest scaffolds is shown in the center of each circle. Repeats are colorcoded: yellow - LINEs, light green - low complexity, magenta - LTRs, blue - rolling circles, dark green - satellites, orange - simple repeats, red - transposons, turquoise - unclassified repeats.

**Additional file 5. **Somy levels and repeat content for 50 longest scaffolds from the genome assemblies obtained in this study.

**Additional file 6. **Violin plot representation of somy for the 50 longest scaffolds for each genome assembly. Estimated scaffold somy is color-coded. Scaffold IDs and the ratio of median-of-means coverage values to the median genome overage are shown on X and Y axes, respectively. Bar plot shows the median coverage value and interquartile range.

**Additional file 7. **Genome ploidy estimation based on the analysis of heterozygous *k*-mer pairs. Total coverage of *k*-mer pairs and normalized minor *k*-mer coverage are plotted on Y and X axes, respectively.

**Additional file 8. **Examples of command lines used for the genome assembly and downstream analyses.

## Data Availability

Raw sequencing reads were deposited in the NCBI database under BioProject accessions PRJNA949447 and PRJNA543408. The assemblies are deposited in NCBI under the following accession numbers: JASAOP000000000 for *C. thermophila,* JASAOD000000000 for *C. brevicula,* JASAOO000000000 for *Z. costaricensis*, JASAON000000000 for *J. drosophilae,* JASAOC000000000 for *H. samuelpessoai*, JASAOB000000000 for *H. tarakana*, JASAOA000000000 for *V. spadyakhi*, JASANZ000000000 for *S. podlipaevi*, JASAOM000000000 for *B. barvae*, JASANY000000000 for *T. avium*, JASANX000000000 for *T. scelopori*, JASANW000000000 for *T. boissoni*, JASANV000000000 for *T. mega*, JASANU000000000 for *T. platydactyli*, JASAOL000000000 for *W. rigidus*, JASANT000000000 for *W. collosoma*, JASANS000000000 for *Wallacemonas* sp. 195SL, JASANR000000000 for *Wallacemonas* sp. MBr04, JASANQ000000000 for *Wallacemonas* sp. Wsd, JASANP000000000 for *Wallacemonas* sp. Trypx, and JAKVQF000000000 for *O. modryi*. All other data generated or analyzed during this study are included in this published article and its supplementary information files.
