# Supplementary material for: Shining the spotlight on the neglected: new high-quality genome assemblies as a gateway to understanding the evolution of Trypanosomatidae
Source: BMC Genomics. 2023 Aug 21;24:471. doi: 10.1186/s12864-023-09591-z (PMC10441713; doi:10.1186/s12864-023-09591-z)
Supplement: Supplementary file 2 — Additional file 2. Blobplots showing the state of genome assemblies before and after decontamination for the following species: Borovskyia barvae (panel A), Obscuromonas modryi (B), Trypanosoma avium (C), Trypanosoma boissoni (D), Trypanosoma mega (E), Trypanosoma platydactyli (F), and Trypanosoma scelopori (G). [file 12864_2023_9591_MOESM2_ESM.pdf]

Additional file 2. Blobplots showing the state of genome assemblies before and after decontamination for the following species: *Borovskyia barvae* (panel A), *Obscuromonas modryi* (B), *Trypanosoma avium* (C), *Trypanosoma boissoni* (D), *Trypanosoma mega* (E), *Trypanosoma platydactyli* (F), and *Trypanosoma scelopori* (G).

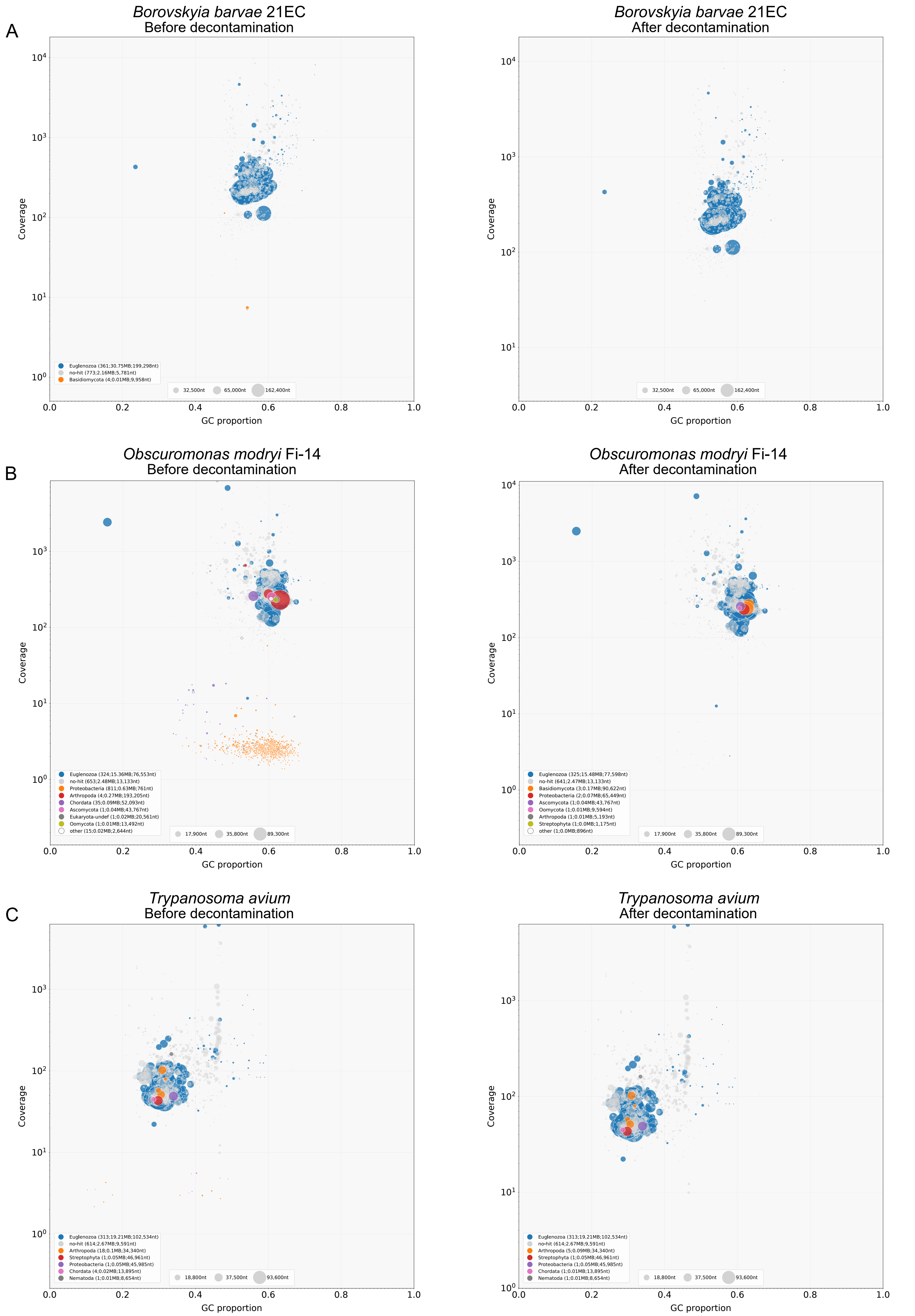

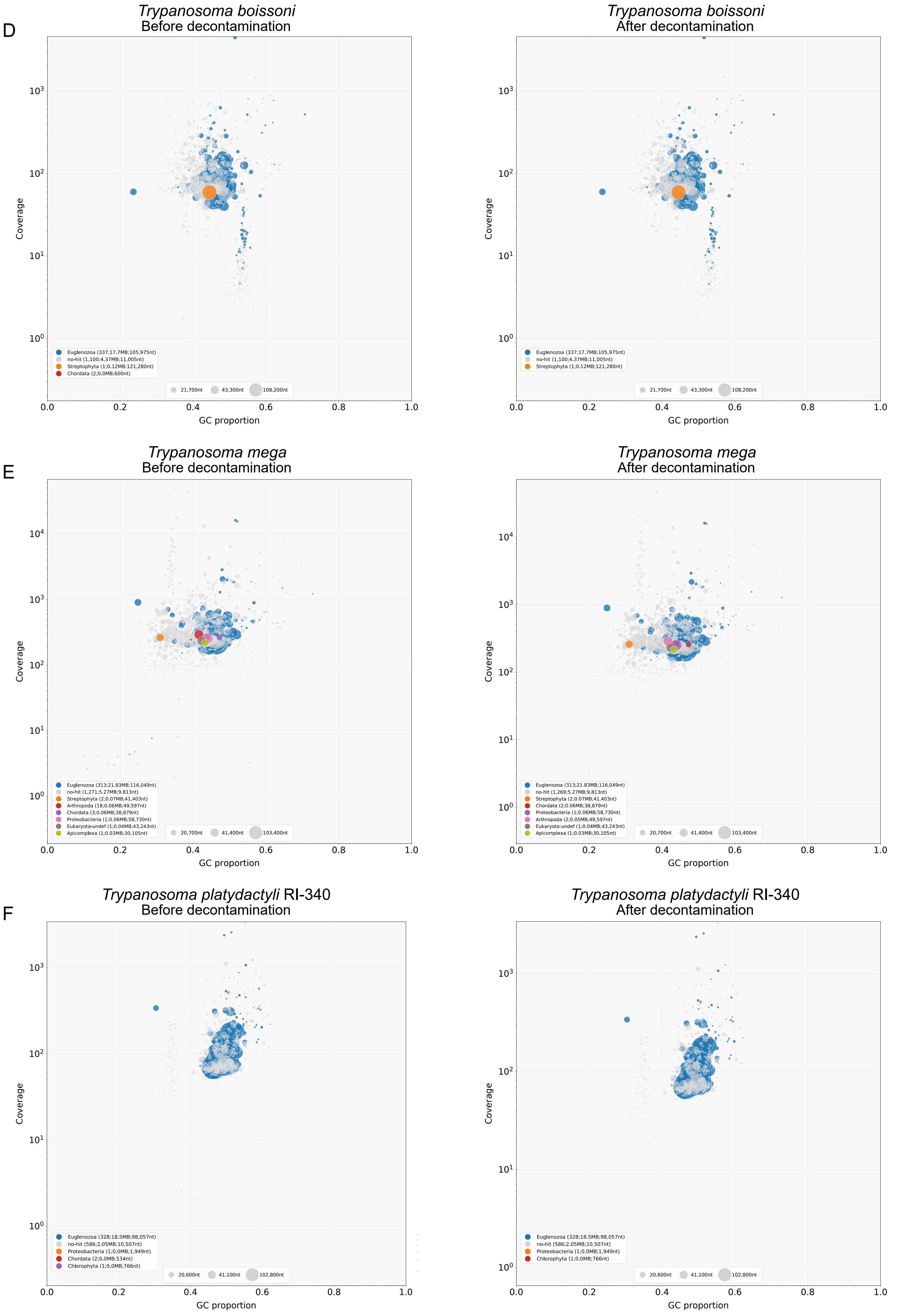
D
Trypanosoma boissoni
Before decontamination
Coverage
GC proportion
Euglenozoa (337;17.7MB;105,975nt)
no-hit (1,100;4.37MB;11,005nt)
Streptophyta (1;0.12MB;121,280nt)
Chordata (2;0.0MB;600nt)
21,700nt
43,300nt
108,200nt
Trypanosoma boissoni
After decontamination
Coverage
GC proportion
Euglenozoa (337;17.7MB;105,975nt)
no-hit (1,100;4.37MB;11,005nt)
Streptophyta (1;0.12MB;121,280nt)
21,700nt
43,300nt
108,200nt
E
Trypanosoma mega
Before decontamination
Coverage
GC proportion
Euglenozoa (313;21.83MB;116,049nt)
no-hit (1,271;5.27MB;9,813nt)
Streptophyta (2;0.07MB;41,403nt)
Arthropoda (18;0.06MB;49,597nt)
Chordata (3;0.06MB;38,879nt)
Proteobacteria (1;0.06MB;58,730nt)
Eukaryota-undef (1;0.04MB;43,243nt)
Apicomplexa (1;0.03MB;30,105nt)
20,700nt
41,400nt
103,400nt
Trypanosoma mega
After decontamination
Coverage
GC proportion
Euglenozoa (313;21.83MB;116,049nt)
no-hit (1,269;5.27MB;9,813nt)
Streptophyta (2;0.07MB;41,403nt)
Chordata (2;0.06MB;38,879nt)
Proteobacteria (1;0.06MB;58,730nt)
Arthropoda (2;0.05MB;49,597nt)
Eukaryota-undef (1;0.04MB;43,243nt)
Apicomplexa (1;0.03MB;30,105nt)
20,700nt
41,400nt
103,400nt
F
Trypanosoma platydactyli RI-340
Before decontamination
Coverage
GC proportion
Euglenozoa (328;18.5MB;98,057nt)
no-hit (586;2.05MB;10,507nt)
Proteobacteria (1;0.0MB;1,949nt)
Chordata (2;0.0MB;534nt)
Chlorophyta (1;0.0MB;766nt)
20,600nt
41,100nt
102,800nt
Trypanosoma platydactyli RI-340
After decontamination
Coverage
GC proportion
Euglenozoa (328;18.5MB;98,057nt)
no-hit (586;2.05MB;10,507nt)
Proteobacteria (1;0.0MB;1,949nt)
Chlorophyta (1;0.0MB;766nt)
20,600nt
41,100nt
102,800nt

G

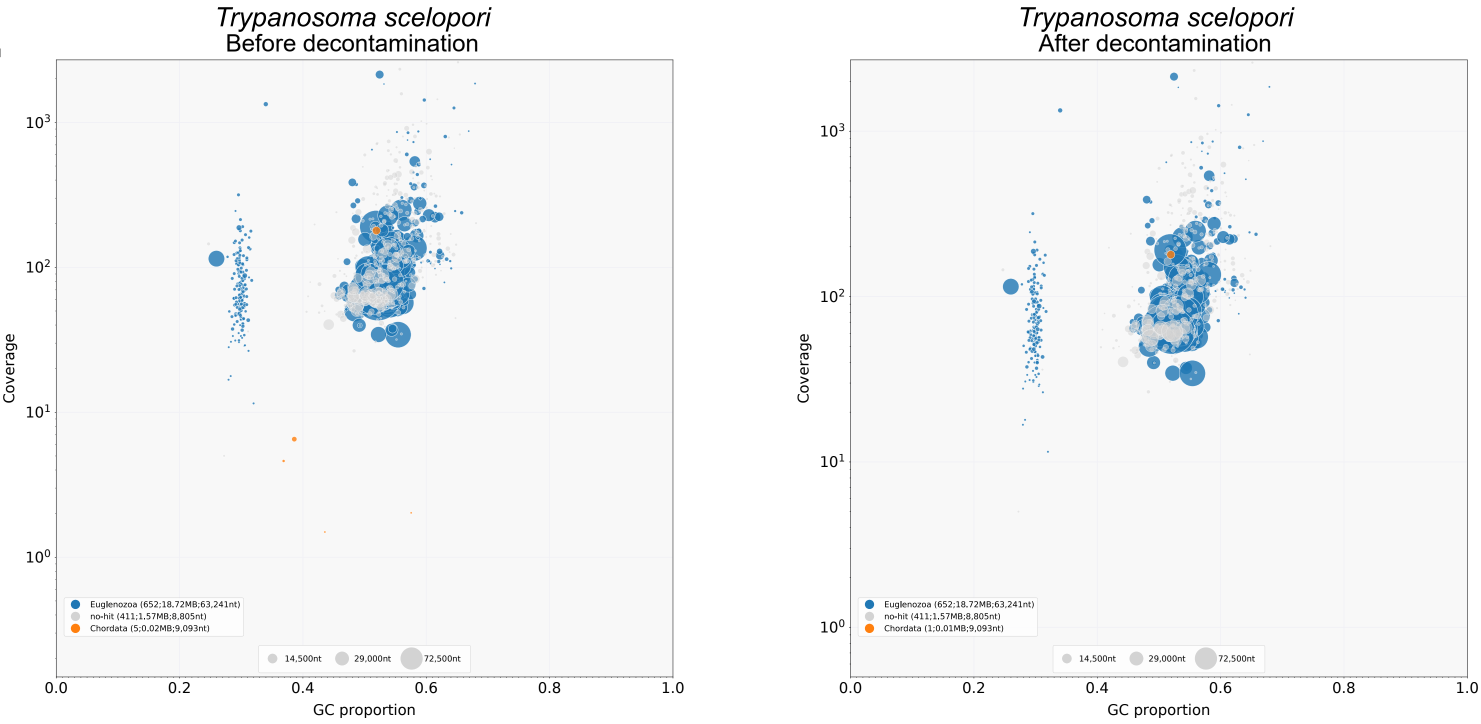
Trypanosoma scelopori
Before decontamination
Coverage
GC proportion
Euglenozoa (652;18.72MB;63,241nt)
no-hit (411;1.57MB;8,805nt)
Chordata (5;0.02MB;9,093nt)
14,500nt
29,000nt
72,500nt
Trypanosoma scelopori
After decontamination
Coverage
GC proportion
Euglenozoa (652;18.72MB;63,241nt)
no-hit (411;1.57MB;8,805nt)
Chordata (1;0.01MB;9,093nt)
14,500nt
29,000nt
72,500nt
