# Supplementary material for: Shining the spotlight on the neglected: new high-quality genome assemblies as a gateway to understanding the evolution of Trypanosomatidae
Source: BMC Genomics. 2023 Aug 21;24:471. doi: 10.1186/s12864-023-09591-z (PMC10441713; doi:10.1186/s12864-023-09591-z)
Supplement: Supplementary file 4 — Additional file 4. J-circos plots showing the distribution of repeats along ten longest scaffolds for each trypanosomatid species. The tracks are the following (from inside out): repeat distribution, GC skew, transcriptomic read mapping, scaffold borders and IDs. Total length of the ten largest scaffolds is shown in the center of each circle. Repeats are colorcoded: yellow - LINEs, light green - low complexity, magenta - LTRs, blue - rolling circles, dark green - satellites, orange - simple repeats, red - transposons, turquoise - unclassified repeats. [file 12864_2023_9591_MOESM4_ESM.pdf]

Additional file 4. J-circos plots showing the distribution of repeats along ten longest scaffolds for each trypanosomatid species. The tracks are the following (from inside out): repeat distribution, GC skew, transcriptomic read mapping, scaffold borders and IDs. Total length of the ten largest scaffolds is shown in the center of each circle. Repeats are color-coded: yellow - LINEs, light green - low complexity, magenta - LTRs, blue - rolling circles, dark green - satellites, orange - simple repeats, red - transposons, turquoise - unclassified repeats.

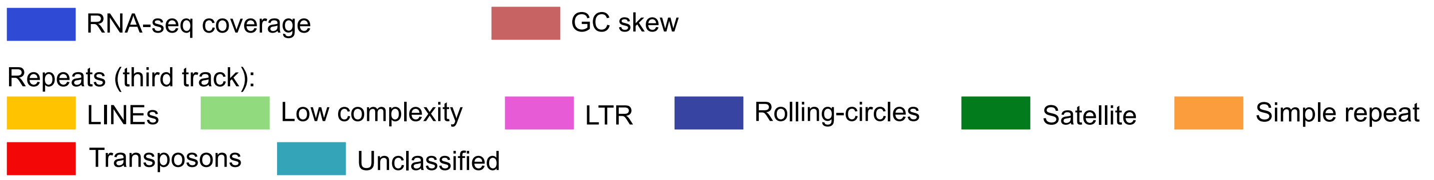

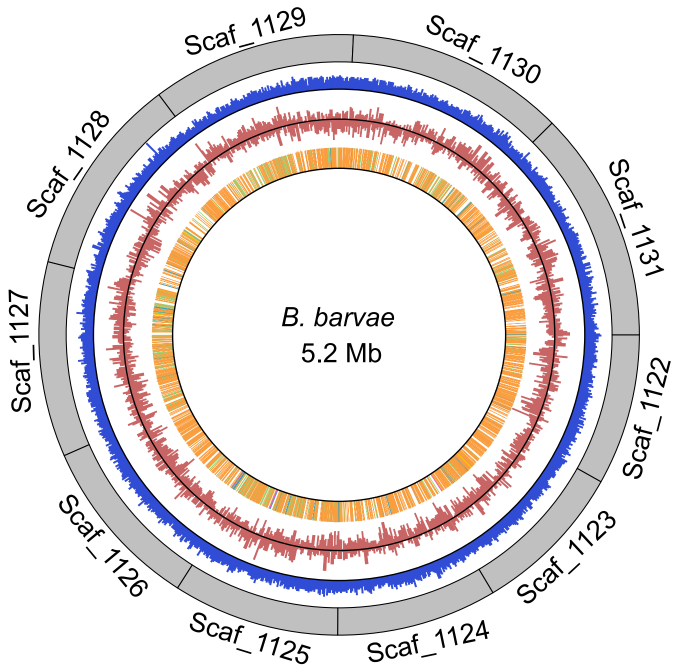

20 LINEs, 444 Low complexity, 6 LTRs, 2,927 Simple repeats, 102 Unclassified
Total repeats: 160,793 bp
Transcribed repeats: 141,807 bp

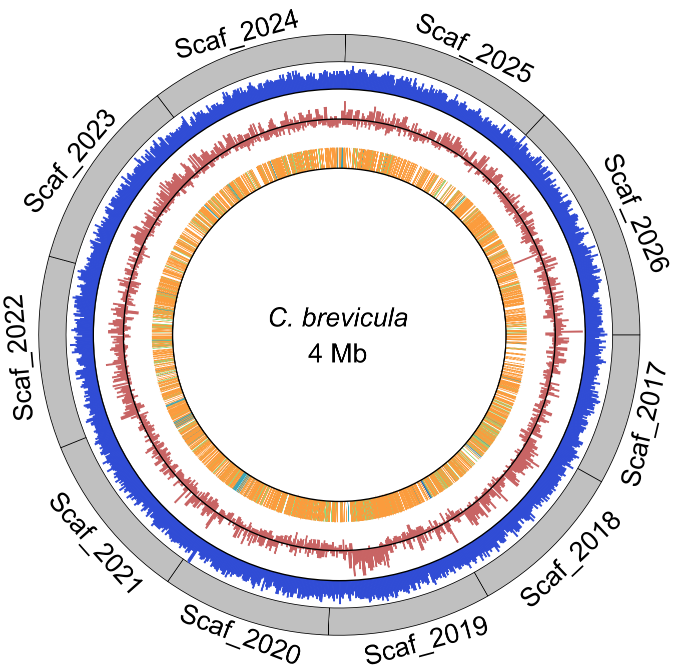

425 Low complexity, 2 LTRs, 1 Rolling-circle, 3,240 Simple repeats, 76 Unclassified
Total repeats: 175,233 bp
Transcribed repeats: 164,367 bp

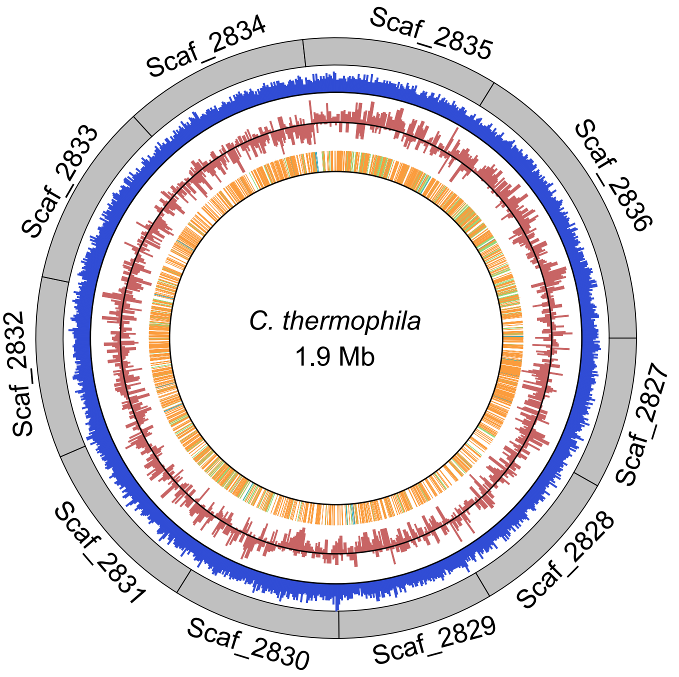

236 Low complexity, 1,755 Simple repeats, 26 Unclassified
Total repeats: 82,256 bp
Transcribed repeats: 79,441 bp

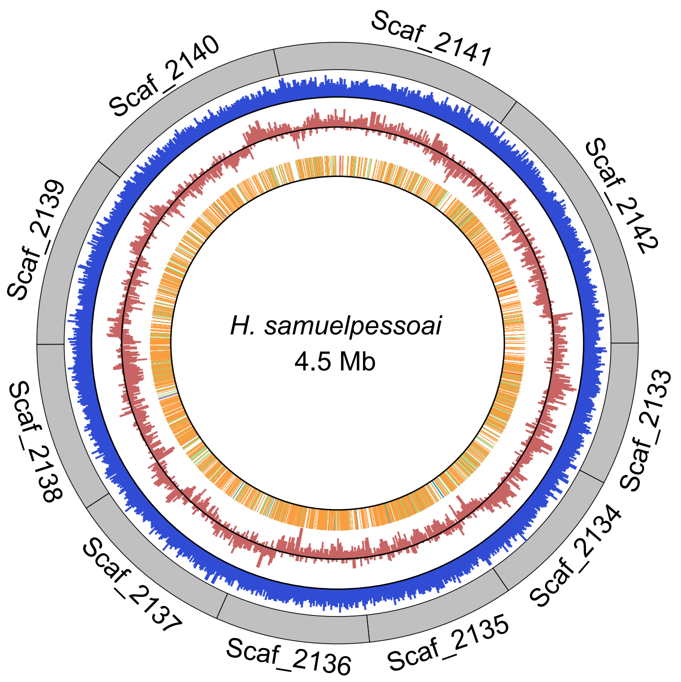

411 Low complexity, 4 LTRs, 2,349 Simple repeats, 7 Transposons, 22 Unclassified
Total repeats: 114,623 bp
Transcribed repeats: 99,717 bp

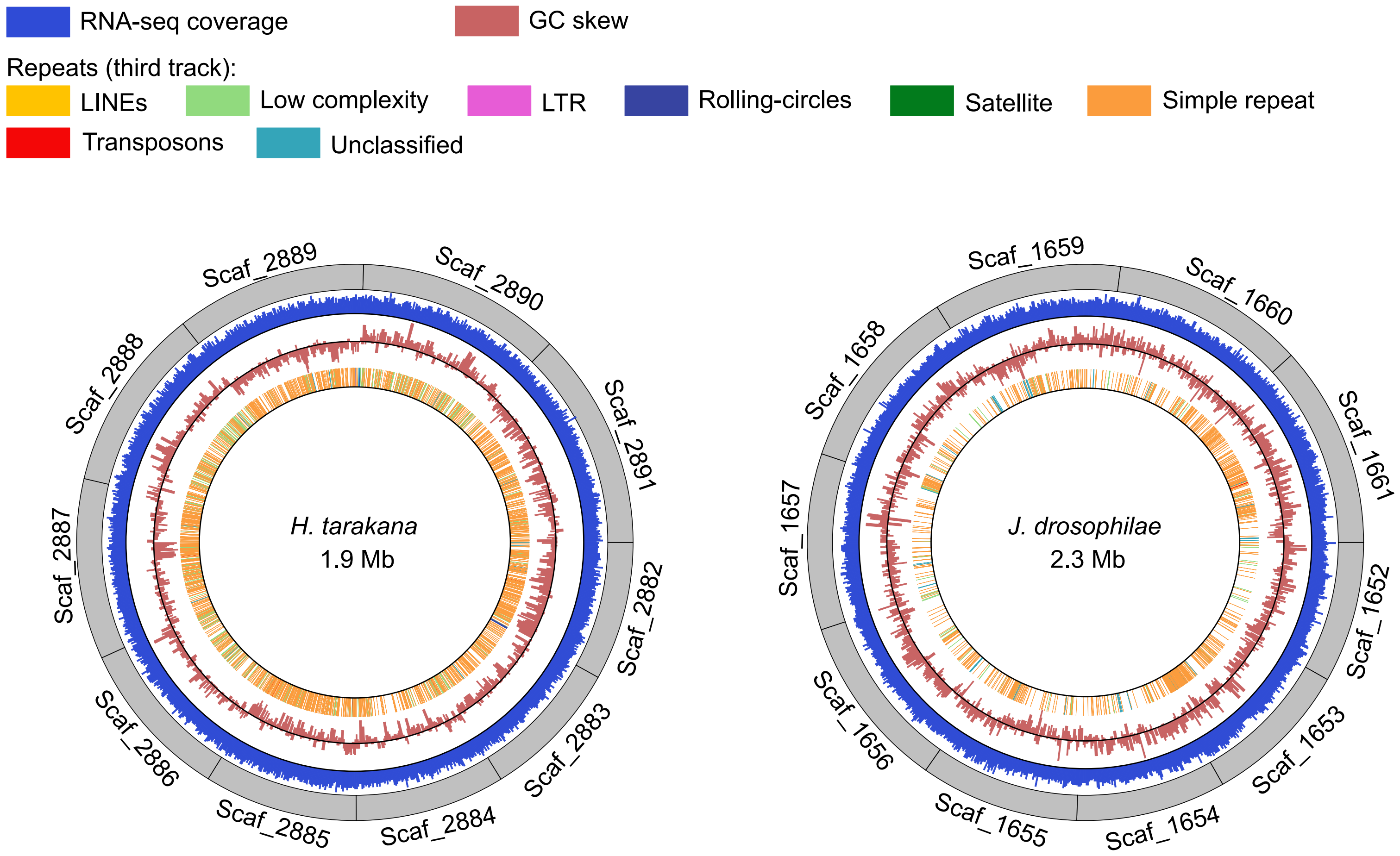

286 Low complexity, 1 LTR, 2 Rolling-circles, 1,970 Simple repeats, 8 Transposons, 20 Unclassified
Total repeats: 93,652 bp
Transcribed repeats: 89,716 bp

71 Low complexity, 892 Simple repeats, 2 Transposons, 51 Unclassified
Total repeats: 49,655 bp
Transcribed repeats: 46,319 bp

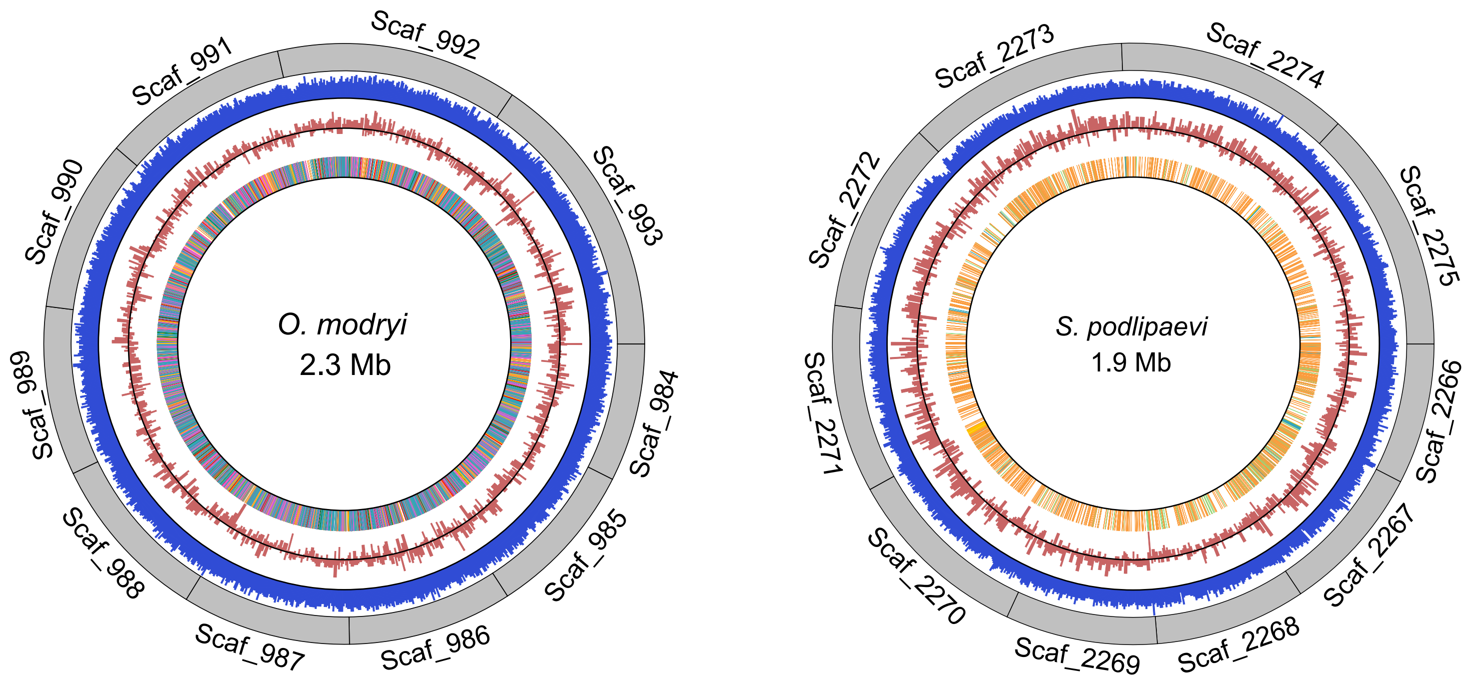

556 LINEs, 23 Low complexity, 1,735 LTRs, 176 Satellites, 3,264 Simple repeats, 472 Transposons, 3,234 Unclassified
Total repeats: 989,415 bp
Transcribed repeats: 988,427 bp

11 LINEs, 235 Low complexity, 1,736 Simple repeats, 2 Transposons, 36 Unclassified
Total repeats: 91,736 bp
Transcribed repeats: 84,527 bp

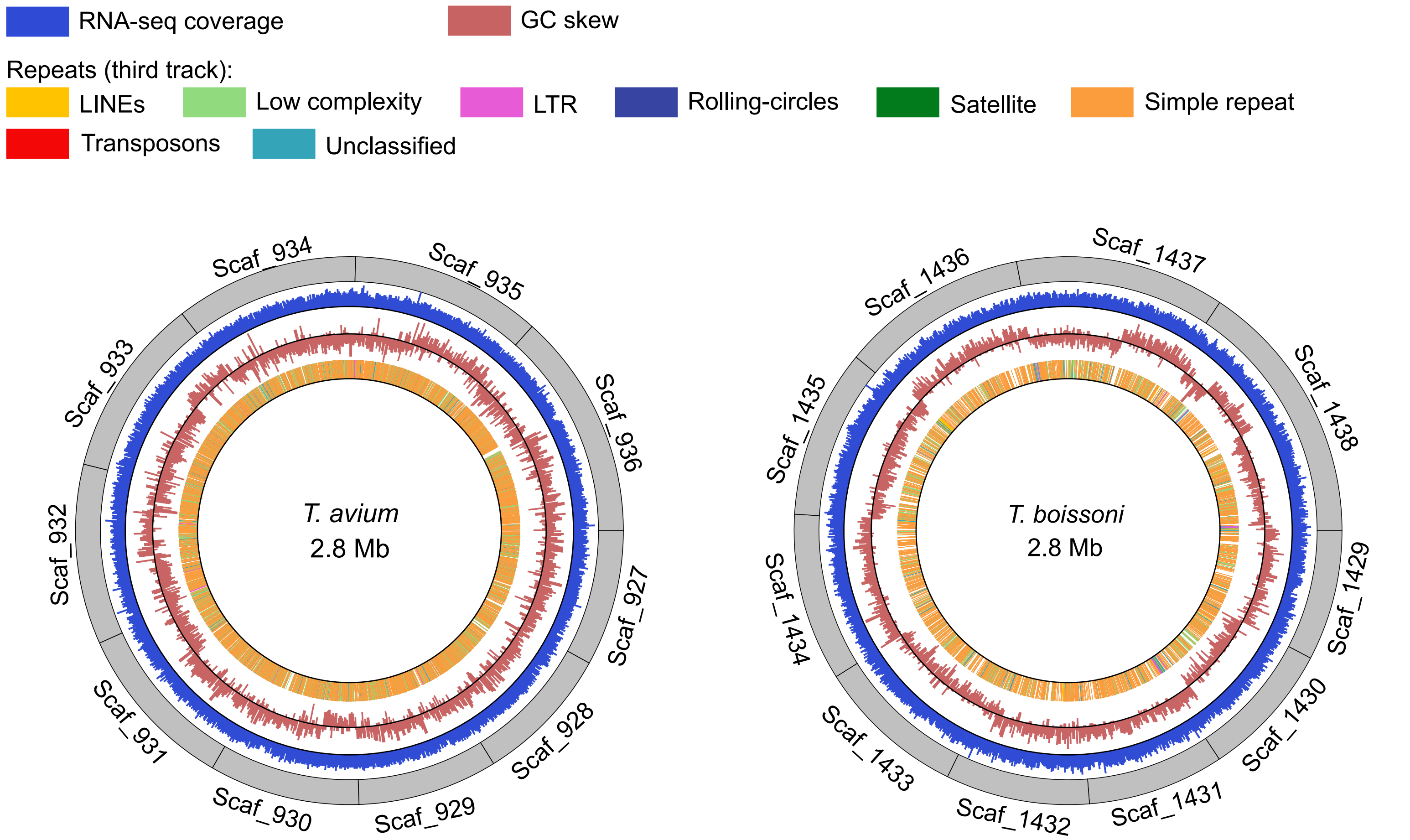

1,485 Low complexity, 37 LTRs, 1 Rolling-circles, 7,787 Simple repeats, 1 Transposons, 241 Unclassified

Total repeats: 436,195 bp

Transcribed repeats: 434,757 bp

567 Low complexity, 30 LINEs, 22 LTRs, 3,165 Simple repeats, 146 Unclassified

Total repeats: 209,605 bp

Transcribed repeats: 207,862 bp

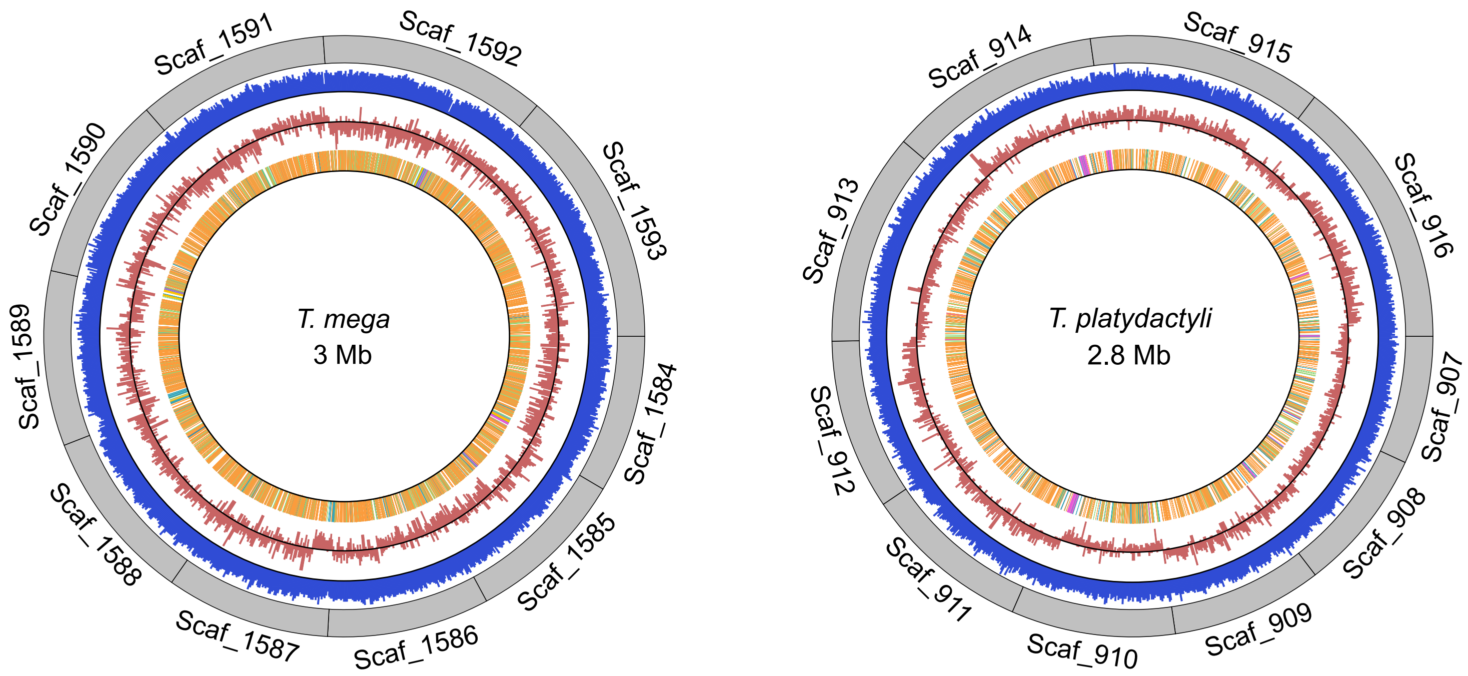

10 LINEs, 886 Low complexity, 22 LTRs, 1 Rolling-circle, 4,110 Simple repeats, 185 Unclassified

Total repeats: 307,412 bp

Transcribed repeats: 287,607 bp

286 Low complexity, 61 LTRs, 1,798 Simple repeats, 206 Unclassified

Total repeats: 171,075 bp

Transcribed repeats: 162,414 bp

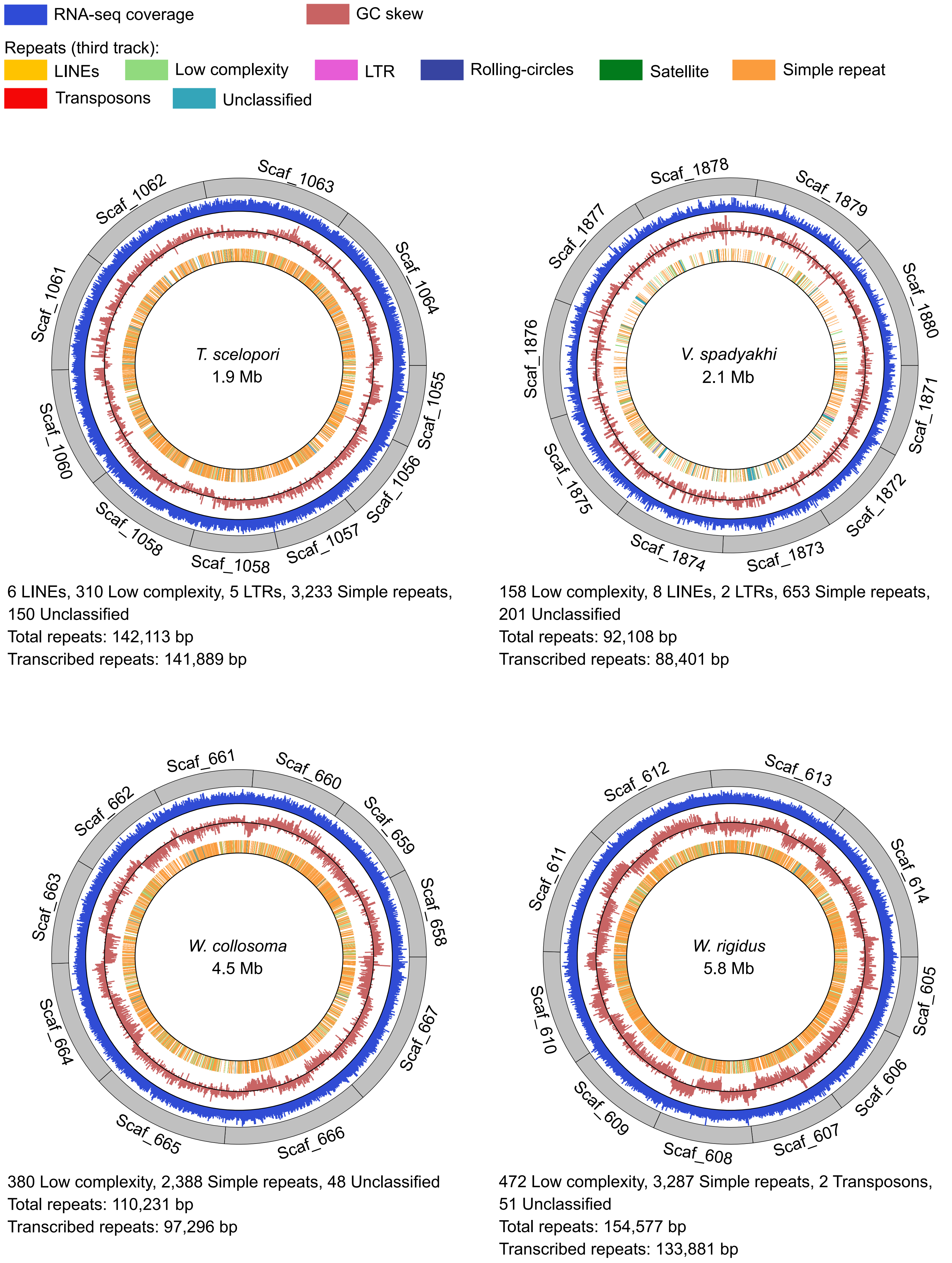

RNA-seq coverage
GC skew
Repeats (third track):
LINEs
Low complexity
LTR
Rolling-circles
Satellite
Simple repeat
Transposons
Unclassified
T. scelopori
1.9 Mb
Scaf_1063
Scaf_1064
Scaf_1055
Scaf_1056
Scaf_1057
Scaf_1058
Scaf_1058
Scaf_1060
Scaf_1061
Scaf_1062
6 LINEs, 310 Low complexity, 5 LTRs, 3,233 Simple repeats, 150 Unclassified
Total repeats: 142,113 bp
Transcribed repeats: 141,889 bp
V. spadyakhi
2.1 Mb
Scaf_1878
Scaf_1879
Scaf_1880
Scaf_1871
Scaf_1872
Scaf_1873
Scaf_1874
Scaf_1875
Scaf_1876
Scaf_1877
158 Low complexity, 8 LINEs, 2 LTRs, 653 Simple repeats, 201 Unclassified
Total repeats: 92,108 bp
Transcribed repeats: 88,401 bp
W. collosoma
4.5 Mb
Scaf_661
Scaf_660
Scaf_659
Scaf_658
Scaf_667
Scaf_666
Scaf_665
Scaf_664
Scaf_663
Scaf_662
380 Low complexity, 2,388 Simple repeats, 48 Unclassified
Total repeats: 110,231 bp
Transcribed repeats: 97,296 bp
W. rigidus
5.8 Mb
Scaf_612
Scaf_613
Scaf_614
Scaf_605
Scaf_606
Scaf_607
Scaf_608
Scaf_609
Scaf_610
Scaf_611
472 Low complexity, 3,287 Simple repeats, 2 Transposons, 51 Unclassified
Total repeats: 154,577 bp
Transcribed repeats: 133,881 bp

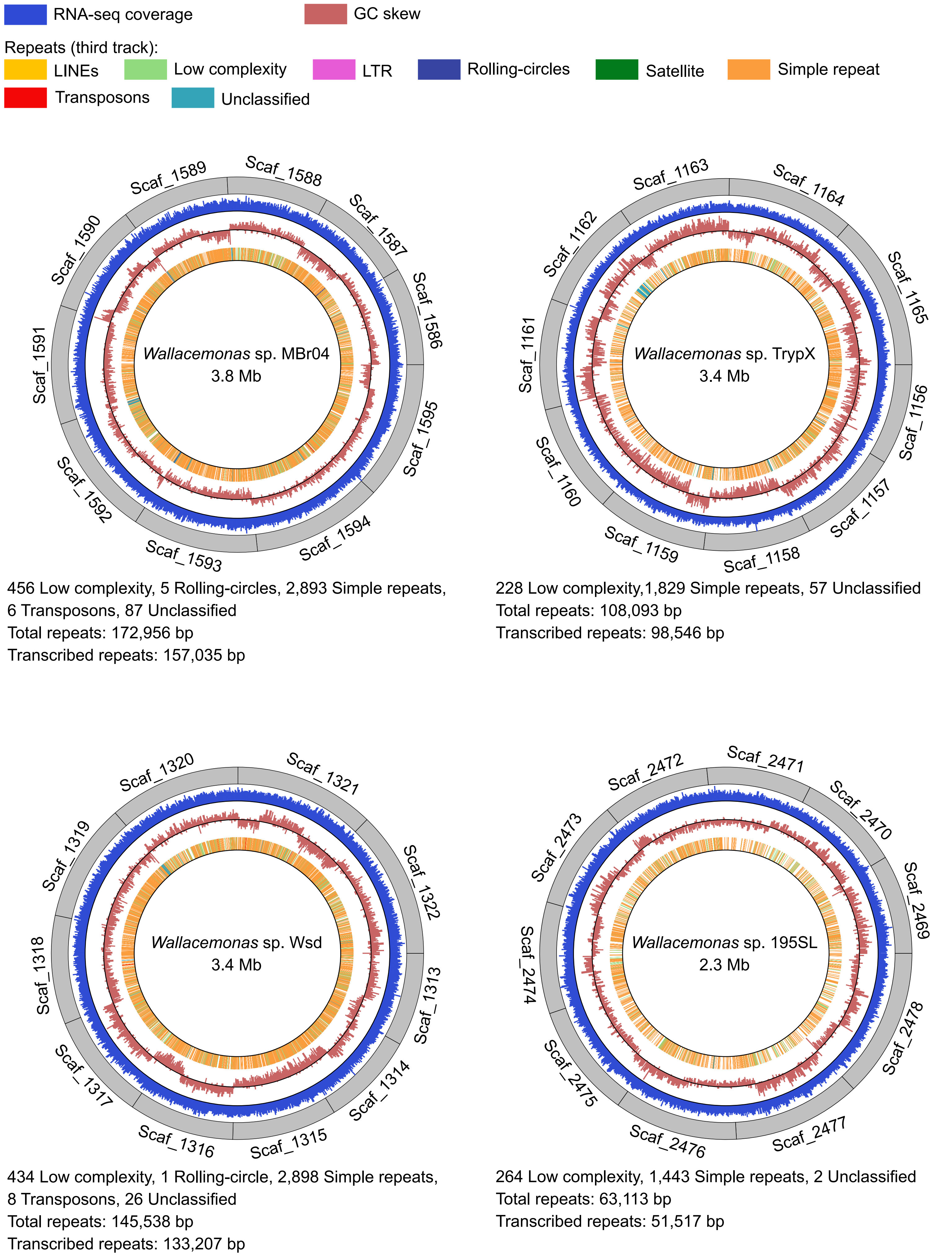
RNA-seq coverage
GC skew
Repeats (third track):
LINEs
Low complexity
LTR
Rolling-circles
Satellite
Simple repeat
Transposons
Unclassified
Scaf_1588
Scaf_1587
Scaf_1586
Scaf_1595
Scaf_1594
Scaf_1593
Scaf_1592
Scaf_1591
Scaf_1590
Scaf_1589
Wallacemonas sp. MBr04
3.8 Mb
456 Low complexity, 5 Rolling-circles, 2,893 Simple repeats,
6 Transposons, 87 Unclassified
Total repeats: 172,956 bp
Transcribed repeats: 157,035 bp
Scaf_1164
Scaf_1165
Scaf_1156
Scaf_1157
Scaf_1158
Scaf_1159
Scaf_1160
Scaf_1161
Scaf_1162
Scaf_1163
Wallacemonas sp. TrypX
3.4 Mb
228 Low complexity,1,829 Simple repeats, 57 Unclassified
Total repeats: 108,093 bp
Transcribed repeats: 98,546 bp
Scaf_1321
Scaf_1322
Scaf_1313
Scaf_1314
Scaf_1315
Scaf_1316
Scaf_1317
Scaf_1318
Scaf_1319
Scaf_1320
Wallacemonas sp. Wsd
3.4 Mb
434 Low complexity, 1 Rolling-circle, 2,898 Simple repeats,
8 Transposons, 26 Unclassified
Total repeats: 145,538 bp
Transcribed repeats: 133,207 bp
Scaf_2471
Scaf_2470
Scaf_2469
Scaf_2478
Scaf_2477
Scaf_2476
Scaf_2475
Scaf_2474
Scaf_2473
Scaf_2472
Wallacemonas sp. 195SL
2.3 Mb
264 Low complexity, 1,443 Simple repeats, 2 Unclassified
Total repeats: 63,113 bp
Transcribed repeats: 51,517 bp

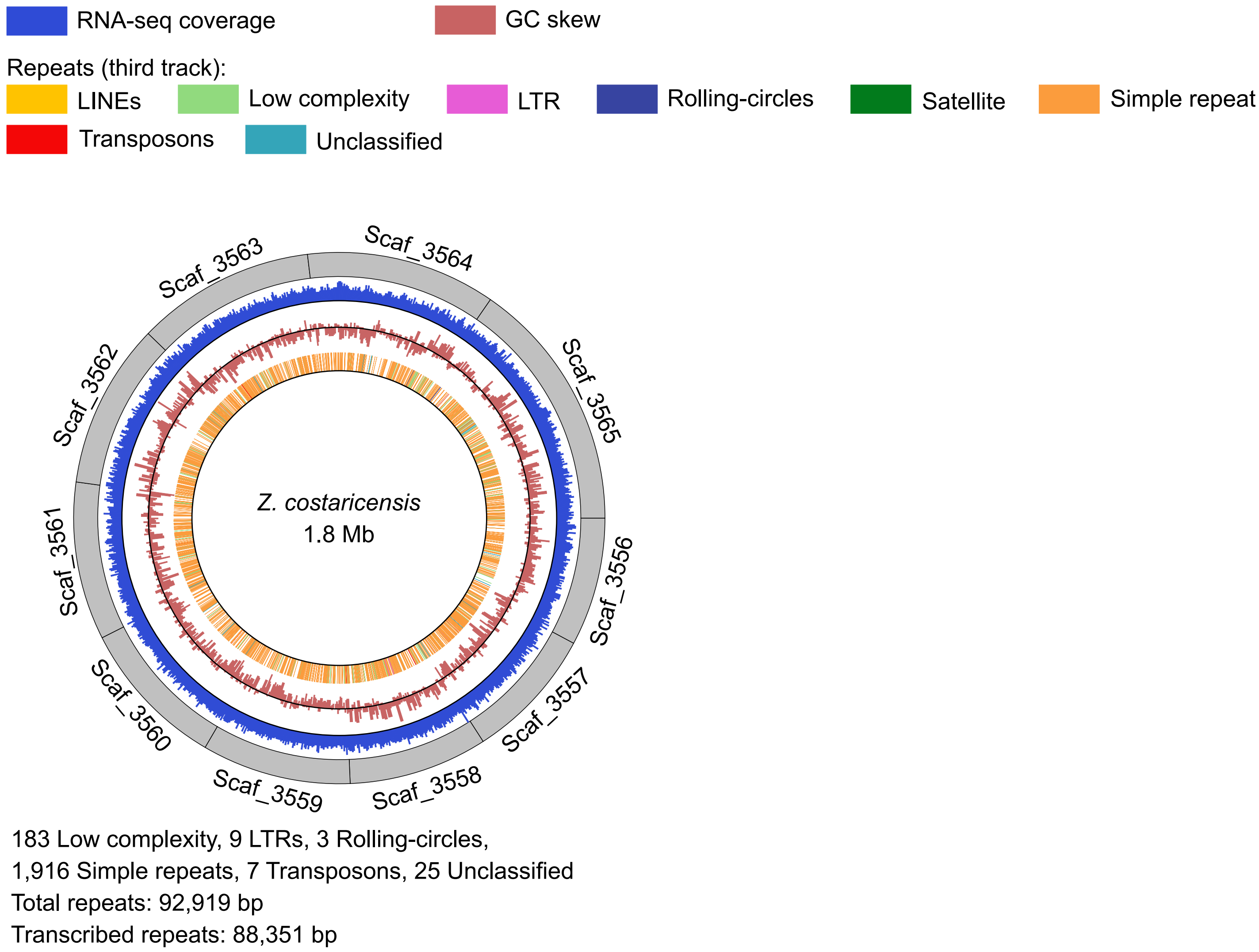
RNA-seq coverage
GC skew
Repeats (third track):
LINEs
Low complexity
LTR
Rolling-circles
Satellite
Simple repeat
Transposons
Unclassified
Scaf_3563
Scaf_3564
Scaf_3565
Scaf_3556
Scaf_3557
Scaf_3558
Scaf_3559
Scaf_3560
Scaf_3561
Scaf_3562
Z. costaricensis
1.8 Mb
183 Low complexity, 9 LTRs, 3 Rolling-circles,
1,916 Simple repeats, 7 Transposons, 25 Unclassified
Total repeats: 92,919 bp
Transcribed repeats: 88,351 bp
