# Supplementary material for: Shining the spotlight on the neglected: new high-quality genome assemblies as a gateway to understanding the evolution of Trypanosomatidae
Source: BMC Genomics. 2023 Aug 21;24:471. doi: 10.1186/s12864-023-09591-z (PMC10441713; doi:10.1186/s12864-023-09591-z)
Supplement: Supplementary file 6 — Additional file 6. Violin plot representation of somy for the 50 longest scaffolds for each genome assembly. Estimated scaffold somy is color-coded. Scaffold IDs and the ratio of median-of-means coverage values to the median genome overage are shown on X and Y axes, respectively. Bar plot shows the median coverage value and interquartile range. [file 12864_2023_9591_MOESM6_ESM.pdf]

Additional file 6. Violin plot representation of somy for the 50 longest scaffolds for each genome assembly. Estimated scaffold somy is color-coded. Scaffold IDs and the ratio of median-of-means coverage values to the median genome overage are shown on X and Y axes, respectively. Bar plot shows the median coverage value and interquartile range.

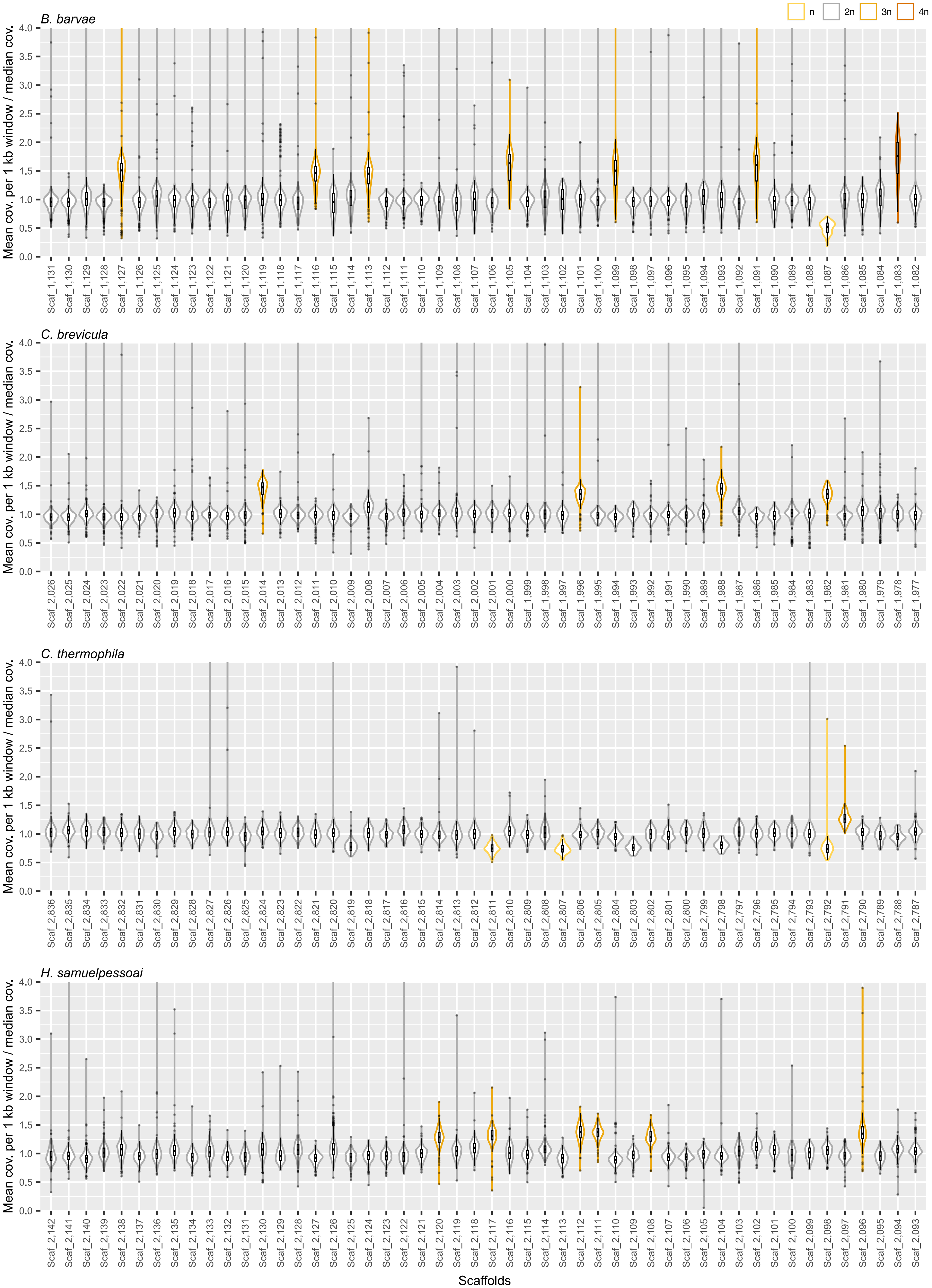

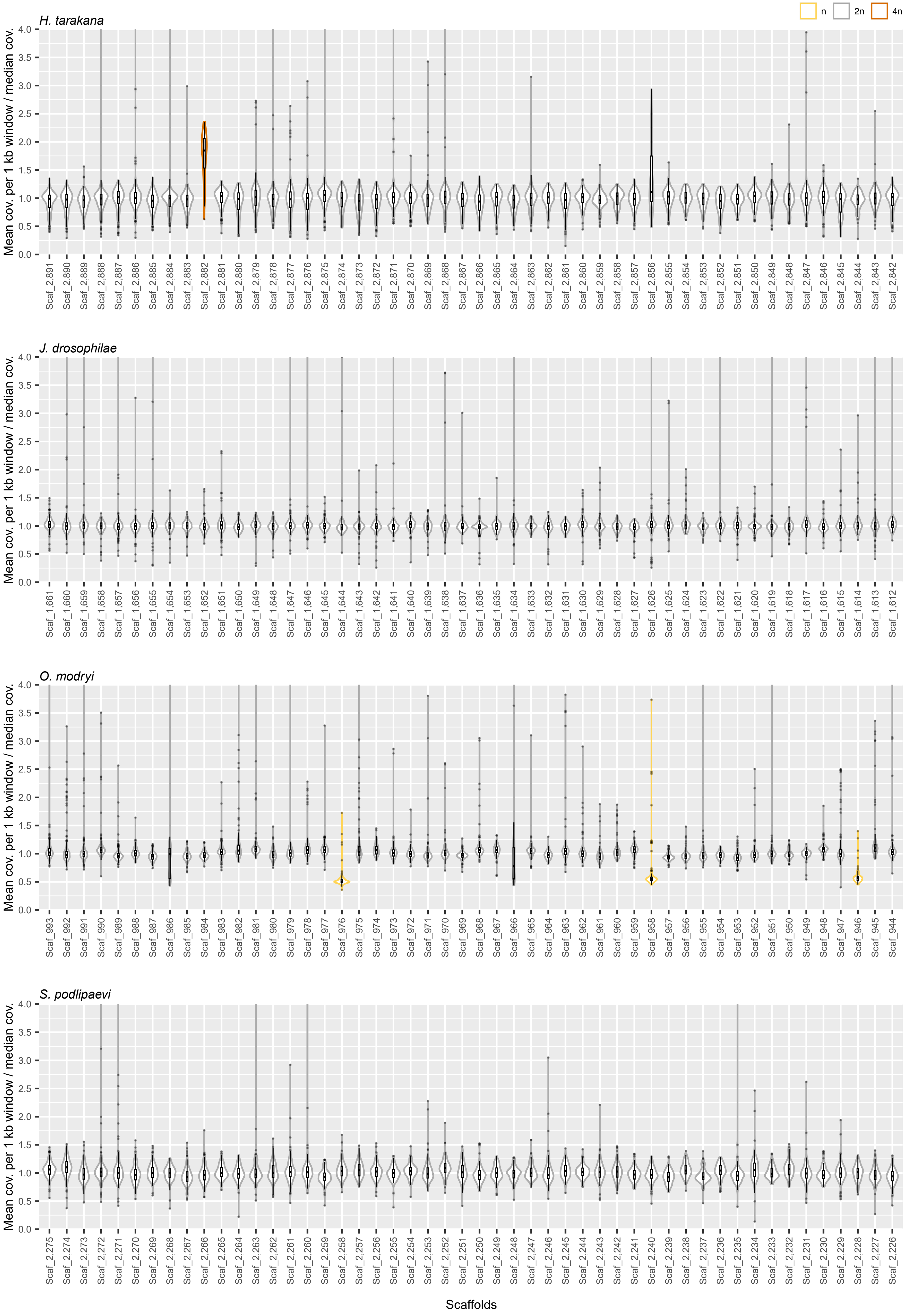
n
2n
4n
H. tarakana
Mean cov. per 1 kb window / median cov.
0.0 0.5 1.0 1.5 2.0 2.5 3.0 3.5 4.0
Scaf_2,891 Scaf_2,890 Scaf_2,889 Scaf_2,888 Scaf_2,887 Scaf_2,886 Scaf_2,885 Scaf_2,884 Scaf_2,883 Scaf_2,882 Scaf_2,881 Scaf_2,880 Scaf_2,879 Scaf_2,878 Scaf_2,877 Scaf_2,876 Scaf_2,875 Scaf_2,874 Scaf_2,873 Scaf_2,872 Scaf_2,871 Scaf_2,870 Scaf_2,869 Scaf_2,868 Scaf_2,867 Scaf_2,866 Scaf_2,865 Scaf_2,864 Scaf_2,863 Scaf_2,862 Scaf_2,861 Scaf_2,860 Scaf_2,859 Scaf_2,858 Scaf_2,857 Scaf_2,856 Scaf_2,855 Scaf_2,854 Scaf_2,853 Scaf_2,852 Scaf_2,851 Scaf_2,850 Scaf_2,849 Scaf_2,848 Scaf_2,847 Scaf_2,846 Scaf_2,845 Scaf_2,844 Scaf_2,843 Scaf_2,842
J. drosophilae
Mean cov. per 1 kb window / median cov.
0.0 0.5 1.0 1.5 2.0 2.5 3.0 3.5 4.0
Scaf_1,661 Scaf_1,660 Scaf_1,659 Scaf_1,658 Scaf_1,657 Scaf_1,656 Scaf_1,655 Scaf_1,654 Scaf_1,653 Scaf_1,652 Scaf_1,651 Scaf_1,650 Scaf_1,649 Scaf_1,648 Scaf_1,647 Scaf_1,646 Scaf_1,645 Scaf_1,644 Scaf_1,643 Scaf_1,642 Scaf_1,641 Scaf_1,640 Scaf_1,639 Scaf_1,638 Scaf_1,637 Scaf_1,636 Scaf_1,635 Scaf_1,634 Scaf_1,633 Scaf_1,632 Scaf_1,631 Scaf_1,630 Scaf_1,629 Scaf_1,628 Scaf_1,627 Scaf_1,626 Scaf_1,625 Scaf_1,624 Scaf_1,623 Scaf_1,622 Scaf_1,621 Scaf_1,620 Scaf_1,619 Scaf_1,618 Scaf_1,617 Scaf_1,616 Scaf_1,615 Scaf_1,614 Scaf_1,613 Scaf_1,612
O. modryi
Mean cov. per 1 kb window / median cov.
0.0 0.5 1.0 1.5 2.0 2.5 3.0 3.5 4.0
Scaf_993 Scaf_992 Scaf_991 Scaf_990 Scaf_989 Scaf_988 Scaf_987 Scaf_986 Scaf_985 Scaf_984 Scaf_983 Scaf_982 Scaf_981 Scaf_980 Scaf_979 Scaf_978 Scaf_977 Scaf_976 Scaf_975 Scaf_974 Scaf_973 Scaf_972 Scaf_971 Scaf_970 Scaf_969 Scaf_968 Scaf_967 Scaf_966 Scaf_965 Scaf_964 Scaf_963 Scaf_962 Scaf_961 Scaf_960 Scaf_959 Scaf_958 Scaf_957 Scaf_956 Scaf_955 Scaf_954 Scaf_953 Scaf_952 Scaf_951 Scaf_950 Scaf_949 Scaf_948 Scaf_947 Scaf_946 Scaf_945 Scaf_944
S. podlipaevi
Mean cov. per 1 kb window / median cov.
0.0 0.5 1.0 1.5 2.0 2.5 3.0 3.5 4.0
Scaf_2,275 Scaf_2,274 Scaf_2,273 Scaf_2,272 Scaf_2,271 Scaf_2,270 Scaf_2,269 Scaf_2,268 Scaf_2,267 Scaf_2,266 Scaf_2,265 Scaf_2,264 Scaf_2,263 Scaf_2,262 Scaf_2,261 Scaf_2,260 Scaf_2,259 Scaf_2,258 Scaf_2,257 Scaf_2,256 Scaf_2,255 Scaf_2,254 Scaf_2,253 Scaf_2,252 Scaf_2,251 Scaf_2,250 Scaf_2,249 Scaf_2,248 Scaf_2,247 Scaf_2,246 Scaf_2,245 Scaf_2,244 Scaf_2,243 Scaf_2,242 Scaf_2,241 Scaf_2,240 Scaf_2,239 Scaf_2,238 Scaf_2,237 Scaf_2,236 Scaf_2,235 Scaf_2,234 Scaf_2,233 Scaf_2,232 Scaf_2,231 Scaf_2,230 Scaf_2,229 Scaf_2,228 Scaf_2,227 Scaf_2,226
Scaffolds

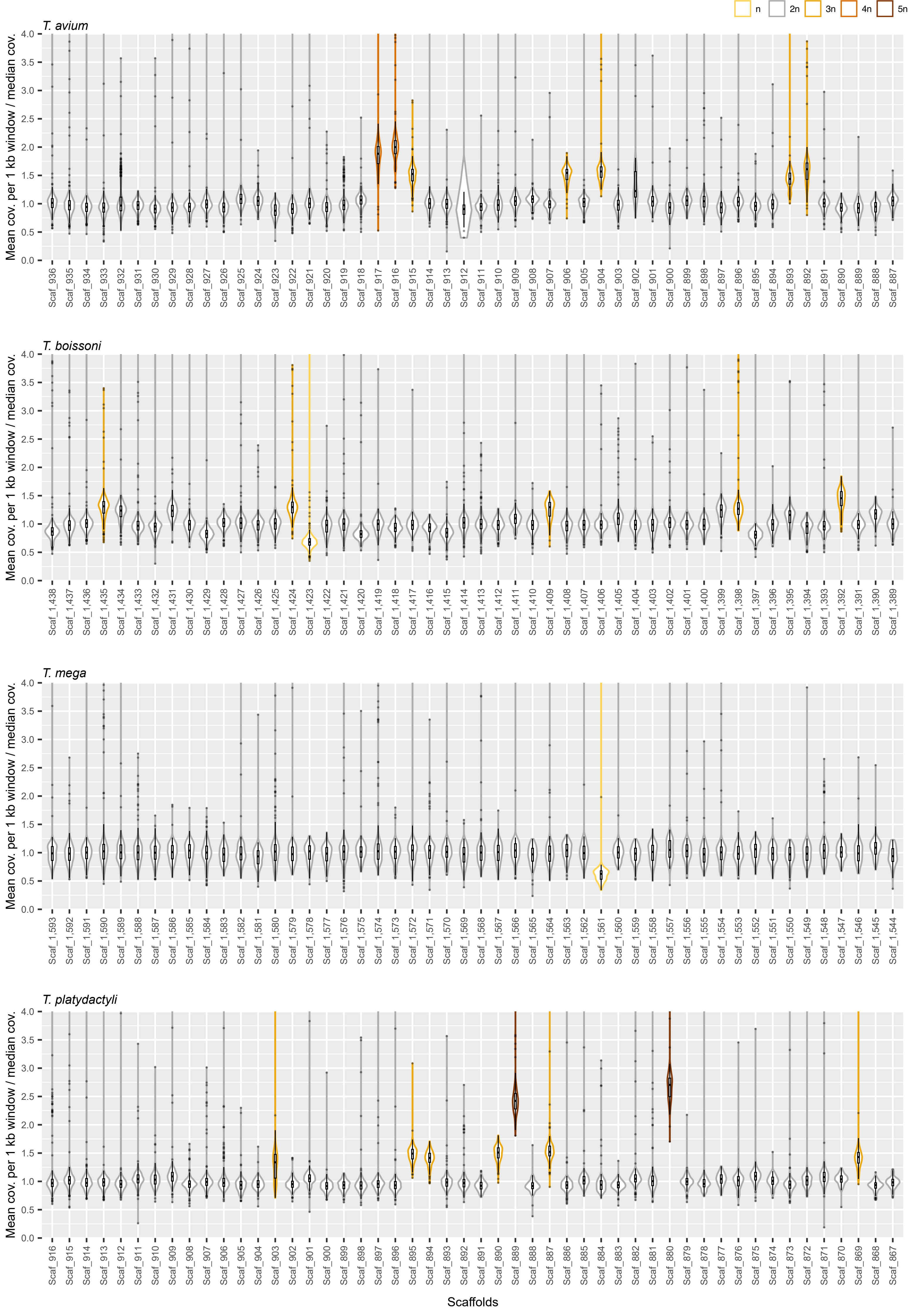
n
2n
3n
4n
5n
T. avium
T. boissoni
T. mega
T. platydactyli
Mean cov. per 1 kb window / median cov.
0.0 0.5 1.0 1.5 2.0 2.5 3.0 3.5 4.0
Scaf_936 Scaf_935 Scaf_934 Scaf_933 Scaf_932 Scaf_931 Scaf_930 Scaf_929 Scaf_928 Scaf_927 Scaf_926 Scaf_925 Scaf_924 Scaf_923 Scaf_922 Scaf_921 Scaf_920 Scaf_919 Scaf_918 Scaf_917 Scaf_916 Scaf_915 Scaf_914 Scaf_913 Scaf_912 Scaf_911 Scaf_910 Scaf_909 Scaf_908 Scaf_907 Scaf_906 Scaf_905 Scaf_904 Scaf_903 Scaf_902 Scaf_901 Scaf_900 Scaf_899 Scaf_898 Scaf_897 Scaf_896 Scaf_895 Scaf_894 Scaf_893 Scaf_892 Scaf_891 Scaf_890 Scaf_889 Scaf_888 Scaf_887
Scaf_1,438 Scaf_1,437 Scaf_1,436 Scaf_1,435 Scaf_1,434 Scaf_1,433 Scaf_1,432 Scaf_1,431 Scaf_1,430 Scaf_1,429 Scaf_1,428 Scaf_1,427 Scaf_1,426 Scaf_1,425 Scaf_1,424 Scaf_1,423 Scaf_1,422 Scaf_1,421 Scaf_1,420 Scaf_1,419 Scaf_1,418 Scaf_1,417 Scaf_1,416 Scaf_1,415 Scaf_1,414 Scaf_1,413 Scaf_1,412 Scaf_1,411 Scaf_1,410 Scaf_1,409 Scaf_1,408 Scaf_1,407 Scaf_1,406 Scaf_1,405 Scaf_1,404 Scaf_1,403 Scaf_1,402 Scaf_1,401 Scaf_1,400 Scaf_1,399 Scaf_1,398 Scaf_1,397 Scaf_1,396 Scaf_1,395 Scaf_1,394 Scaf_1,393 Scaf_1,392 Scaf_1,391 Scaf_1,390 Scaf_1,389
Scaf_1,593 Scaf_1,592 Scaf_1,591 Scaf_1,590 Scaf_1,589 Scaf_1,588 Scaf_1,587 Scaf_1,586 Scaf_1,585 Scaf_1,584 Scaf_1,583 Scaf_1,582 Scaf_1,581 Scaf_1,580 Scaf_1,579 Scaf_1,578 Scaf_1,577 Scaf_1,576 Scaf_1,575 Scaf_1,574 Scaf_1,573 Scaf_1,572 Scaf_1,571 Scaf_1,570 Scaf_1,569 Scaf_1,568 Scaf_1,567 Scaf_1,566 Scaf_1,565 Scaf_1,564 Scaf_1,563 Scaf_1,562 Scaf_1,561 Scaf_1,560 Scaf_1,559 Scaf_1,558 Scaf_1,557 Scaf_1,556 Scaf_1,555 Scaf_1,554 Scaf_1,553 Scaf_1,552 Scaf_1,551 Scaf_1,550 Scaf_1,549 Scaf_1,548 Scaf_1,547 Scaf_1,546 Scaf_1,545 Scaf_1,544
Scaf_916 Scaf_915 Scaf_914 Scaf_913 Scaf_912 Scaf_911 Scaf_910 Scaf_909 Scaf_908 Scaf_907 Scaf_906 Scaf_905 Scaf_904 Scaf_903 Scaf_902 Scaf_901 Scaf_900 Scaf_899 Scaf_898 Scaf_897 Scaf_896 Scaf_895 Scaf_894 Scaf_893 Scaf_892 Scaf_891 Scaf_890 Scaf_889 Scaf_888 Scaf_887 Scaf_886 Scaf_885 Scaf_884 Scaf_883 Scaf_882 Scaf_881 Scaf_880 Scaf_879 Scaf_878 Scaf_877 Scaf_876 Scaf_875 Scaf_874 Scaf_873 Scaf_872 Scaf_871 Scaf_870 Scaf_869 Scaf_868 Scaf_867
Scaffolds

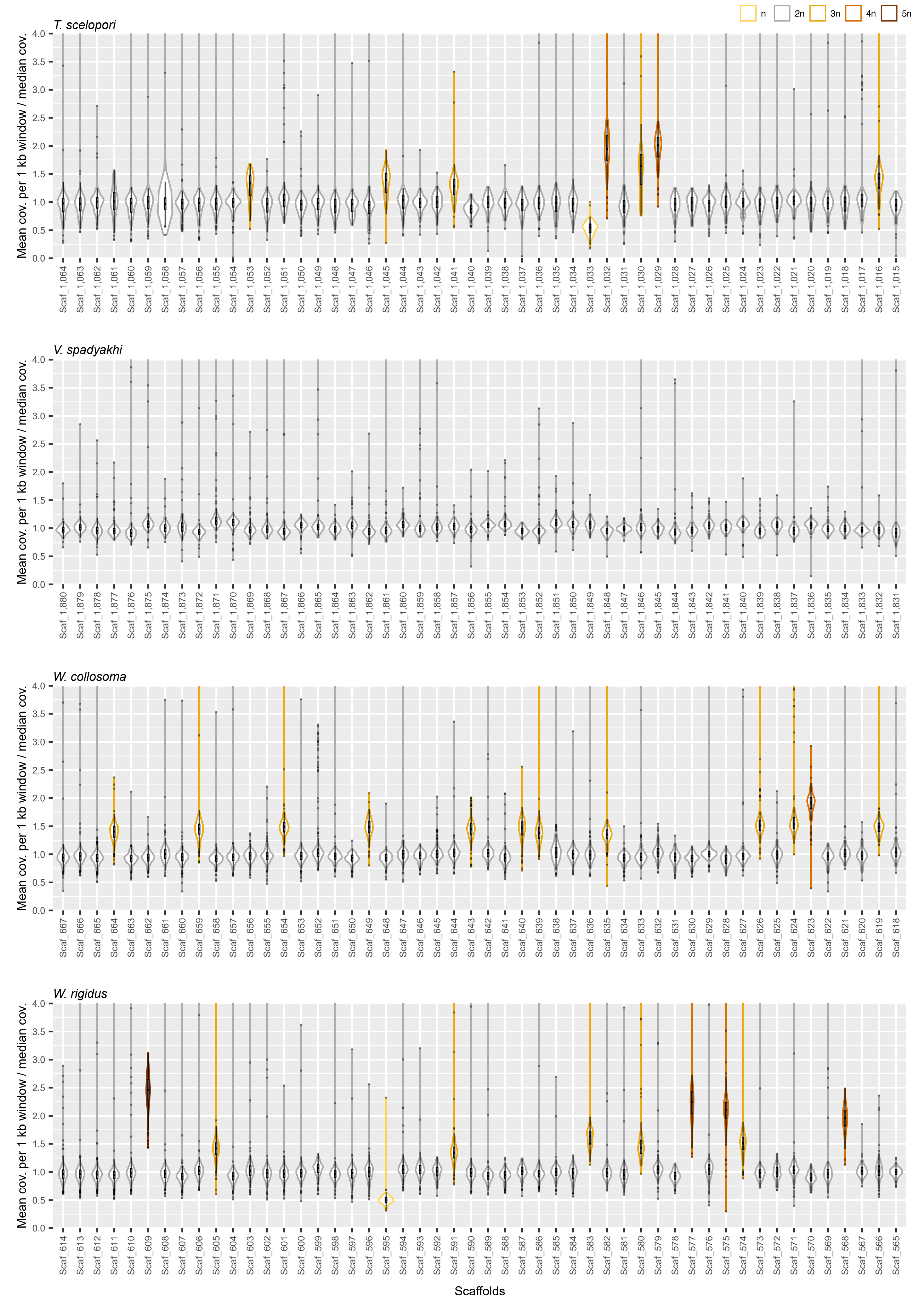
n
2n
3n
4n
5n
T. scelopori
V. spadyakhi
W. collosoma
W. rigidus
Mean cov. per 1 kb window / median cov.
Scaffolds

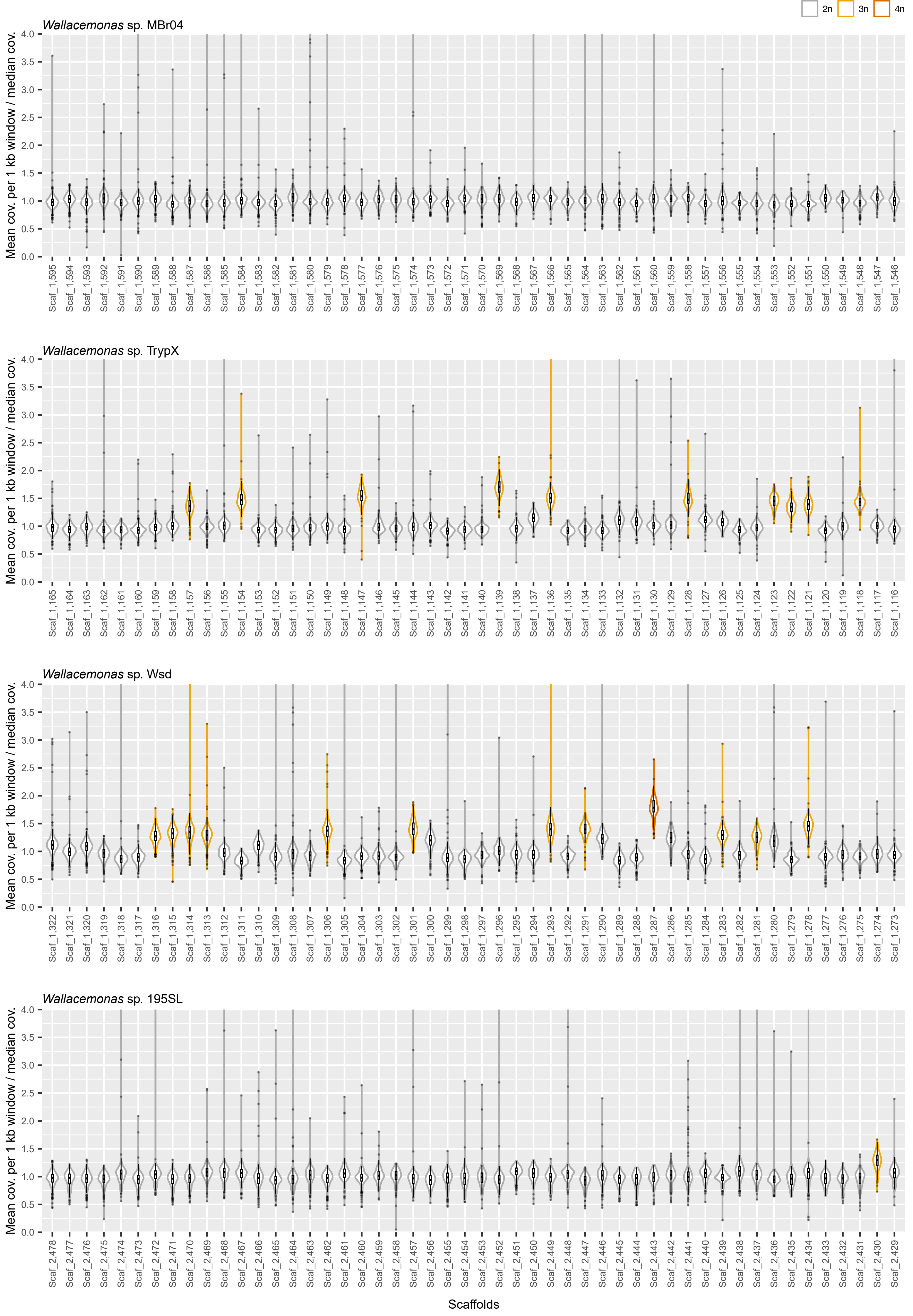
2n
3n
4n
Wallacemonas sp. MBr04
Wallacemonas sp. TrypX
Wallacemonas sp. Wsd
Wallacemonas sp. 195SL
Mean cov. per 1 kb window / median cov.
0.0
0.5
1.0
1.5
2.0
2.5
3.0
3.5
4.0
Scaf_1,595 Scaf_1,594 Scaf_1,593 Scaf_1,592 Scaf_1,591 Scaf_1,590 Scaf_1,589 Scaf_1,588 Scaf_1,587 Scaf_1,586 Scaf_1,585 Scaf_1,584 Scaf_1,583 Scaf_1,582 Scaf_1,581 Scaf_1,580 Scaf_1,579 Scaf_1,578 Scaf_1,577 Scaf_1,576 Scaf_1,575 Scaf_1,574 Scaf_1,573 Scaf_1,572 Scaf_1,571 Scaf_1,570 Scaf_1,569 Scaf_1,568 Scaf_1,567 Scaf_1,566 Scaf_1,565 Scaf_1,564 Scaf_1,563 Scaf_1,562 Scaf_1,561 Scaf_1,560 Scaf_1,559 Scaf_1,558 Scaf_1,557 Scaf_1,556 Scaf_1,555 Scaf_1,554 Scaf_1,553 Scaf_1,552 Scaf_1,551 Scaf_1,550 Scaf_1,549 Scaf_1,548 Scaf_1,547 Scaf_1,546
Scaf_1,165 Scaf_1,164 Scaf_1,163 Scaf_1,162 Scaf_1,161 Scaf_1,160 Scaf_1,159 Scaf_1,158 Scaf_1,157 Scaf_1,156 Scaf_1,155 Scaf_1,154 Scaf_1,153 Scaf_1,152 Scaf_1,151 Scaf_1,150 Scaf_1,149 Scaf_1,148 Scaf_1,147 Scaf_1,146 Scaf_1,145 Scaf_1,144 Scaf_1,143 Scaf_1,142 Scaf_1,141 Scaf_1,140 Scaf_1,139 Scaf_1,138 Scaf_1,137 Scaf_1,136 Scaf_1,135 Scaf_1,134 Scaf_1,133 Scaf_1,132 Scaf_1,131 Scaf_1,130 Scaf_1,129 Scaf_1,128 Scaf_1,127 Scaf_1,126 Scaf_1,125 Scaf_1,124 Scaf_1,123 Scaf_1,122 Scaf_1,121 Scaf_1,120 Scaf_1,119 Scaf_1,118 Scaf_1,117 Scaf_1,116
Scaf_1,322 Scaf_1,321 Scaf_1,320 Scaf_1,319 Scaf_1,318 Scaf_1,317 Scaf_1,316 Scaf_1,315 Scaf_1,314 Scaf_1,313 Scaf_1,312 Scaf_1,311 Scaf_1,310 Scaf_1,309 Scaf_1,308 Scaf_1,307 Scaf_1,306 Scaf_1,305 Scaf_1,304 Scaf_1,303 Scaf_1,302 Scaf_1,301 Scaf_1,300 Scaf_1,299 Scaf_1,298 Scaf_1,297 Scaf_1,296 Scaf_1,295 Scaf_1,294 Scaf_1,293 Scaf_1,292 Scaf_1,291 Scaf_1,290 Scaf_1,289 Scaf_1,288 Scaf_1,287 Scaf_1,286 Scaf_1,285 Scaf_1,284 Scaf_1,283 Scaf_1,282 Scaf_1,281 Scaf_1,280 Scaf_1,279 Scaf_1,278 Scaf_1,277 Scaf_1,276 Scaf_1,275 Scaf_1,274 Scaf_1,273
Scaf_2,478 Scaf_2,477 Scaf_2,476 Scaf_2,475 Scaf_2,474 Scaf_2,473 Scaf_2,472 Scaf_2,471 Scaf_2,470 Scaf_2,469 Scaf_2,468 Scaf_2,467 Scaf_2,466 Scaf_2,465 Scaf_2,464 Scaf_2,463 Scaf_2,462 Scaf_2,461 Scaf_2,460 Scaf_2,459 Scaf_2,458 Scaf_2,457 Scaf_2,456 Scaf_2,455 Scaf_2,454 Scaf_2,453 Scaf_2,452 Scaf_2,451 Scaf_2,450 Scaf_2,449 Scaf_2,448 Scaf_2,447 Scaf_2,446 Scaf_2,445 Scaf_2,444 Scaf_2,443 Scaf_2,442 Scaf_2,441 Scaf_2,440 Scaf_2,439 Scaf_2,438 Scaf_2,437 Scaf_2,436 Scaf_2,435 Scaf_2,434 Scaf_2,433 Scaf_2,432 Scaf_2,431 Scaf_2,430 Scaf_2,429
Scaffolds

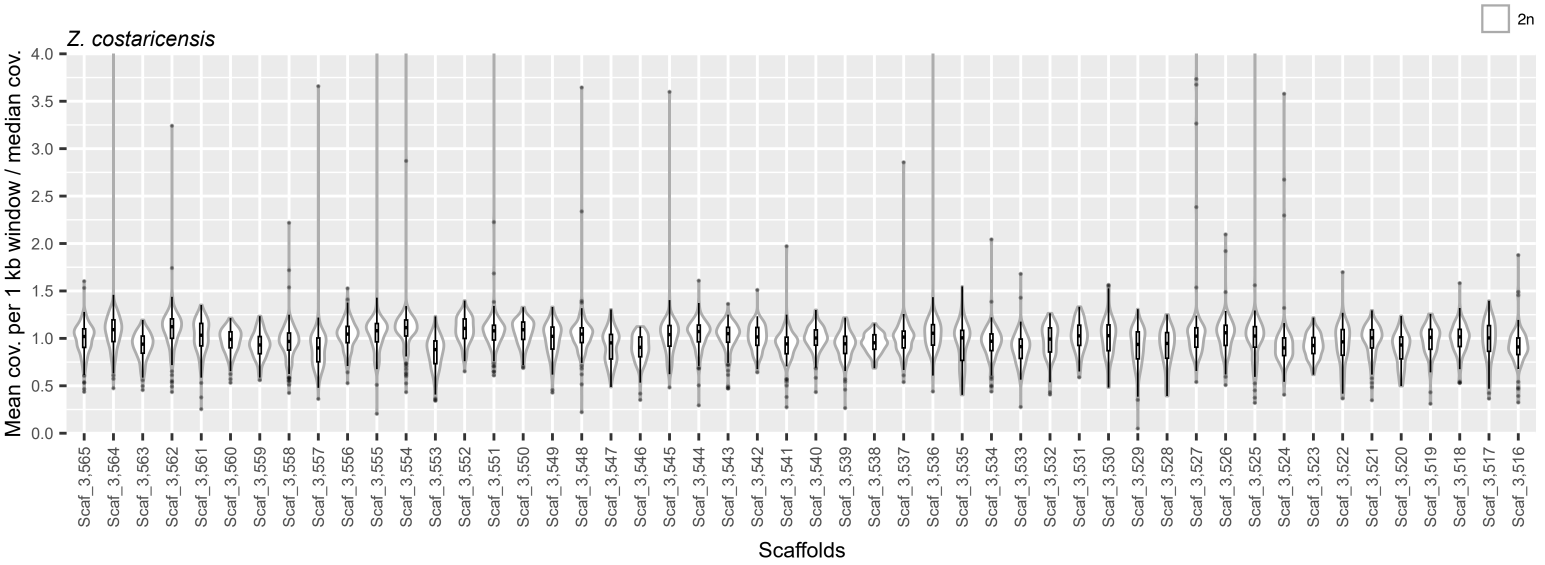

Z. costaricensis
2n
Mean cov. per 1 kb window / median cov.
4.0
3.5
3.0
2.5
2.0
1.5
1.0
0.5
0.0
Scaf_3,565
Scaf_3,564
Scaf_3,563
Scaf_3,562
Scaf_3,561
Scaf_3,560
Scaf_3,559
Scaf_3,558
Scaf_3,557
Scaf_3,556
Scaf_3,555
Scaf_3,554
Scaf_3,553
Scaf_3,552
Scaf_3,551
Scaf_3,550
Scaf_3,549
Scaf_3,548
Scaf_3,547
Scaf_3,546
Scaf_3,545
Scaf_3,544
Scaf_3,543
Scaf_3,542
Scaf_3,541
Scaf_3,540
Scaf_3,539
Scaf_3,538
Scaf_3,537
Scaf_3,536
Scaf_3,535
Scaf_3,534
Scaf_3,533
Scaf_3,532
Scaf_3,531
Scaf_3,530
Scaf_3,529
Scaf_3,528
Scaf_3,527
Scaf_3,526
Scaf_3,525
Scaf_3,524
Scaf_3,523
Scaf_3,522
Scaf_3,521
Scaf_3,520
Scaf_3,519
Scaf_3,518
Scaf_3,517
Scaf_3,516
Scaffolds
