# Supplementary material for: Shining the spotlight on the neglected: new high-quality genome assemblies as a gateway to understanding the evolution of Trypanosomatidae
Source: BMC Genomics. 2023 Aug 21;24:471. doi: 10.1186/s12864-023-09591-z (PMC10441713; doi:10.1186/s12864-023-09591-z)
Supplement: Supplementary file 7 — Additional file 7. Genome ploidy estimation based on the analysis of heterozygous k-mer pairs. Total coverage of k-mer pairs and normalized minor k-mer coverage are plotted on Y and X axes, respectively. [file 12864_2023_9591_MOESM7_ESM.pdf]

Additional file 7. Genome ploidy estimation based on the analysis of heterozygous *k*-mer pairs. Total coverage of *k*-mer pairs and normalized minor *k*-mer coverage are plotted on Y and X axes, respectively.

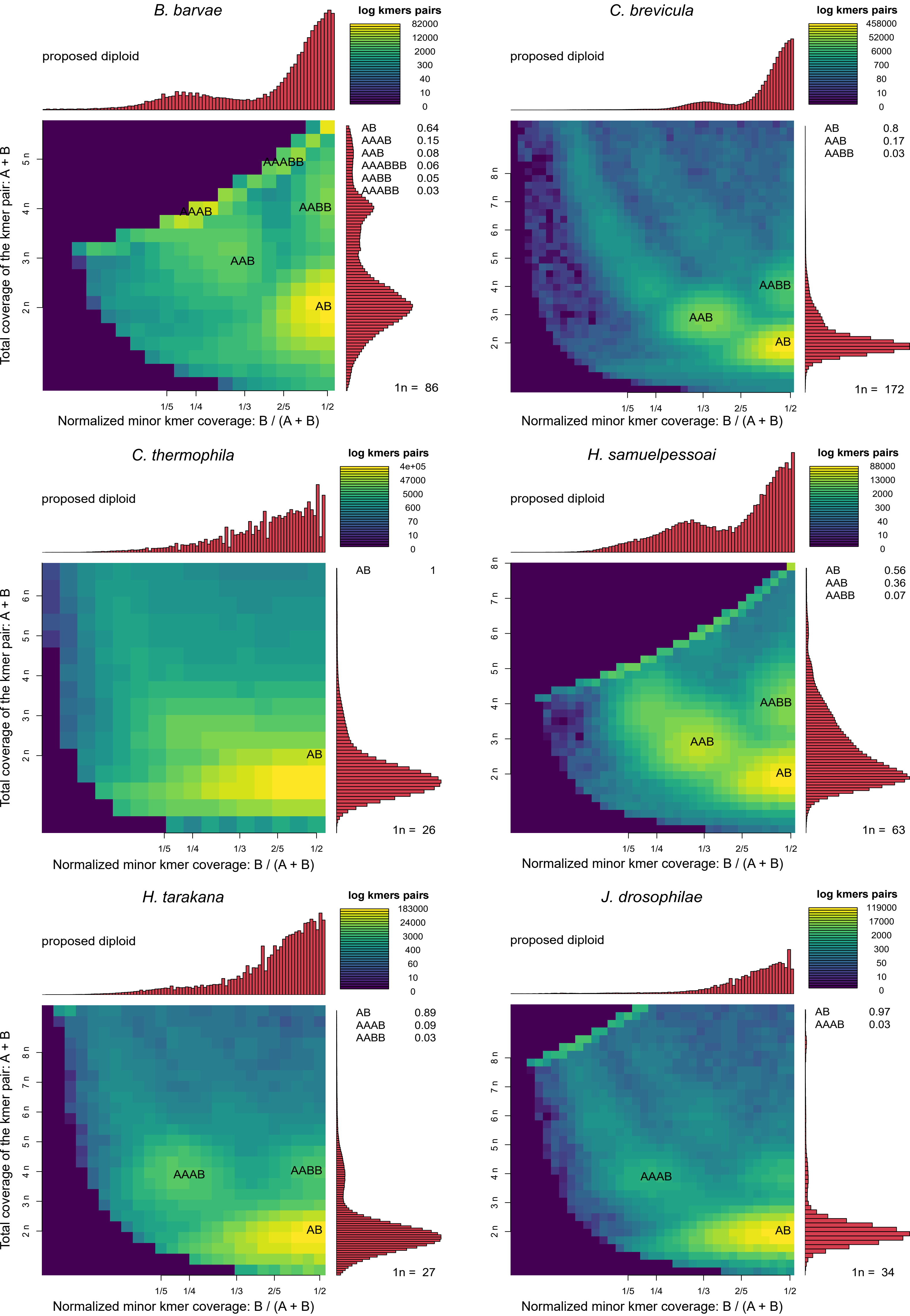

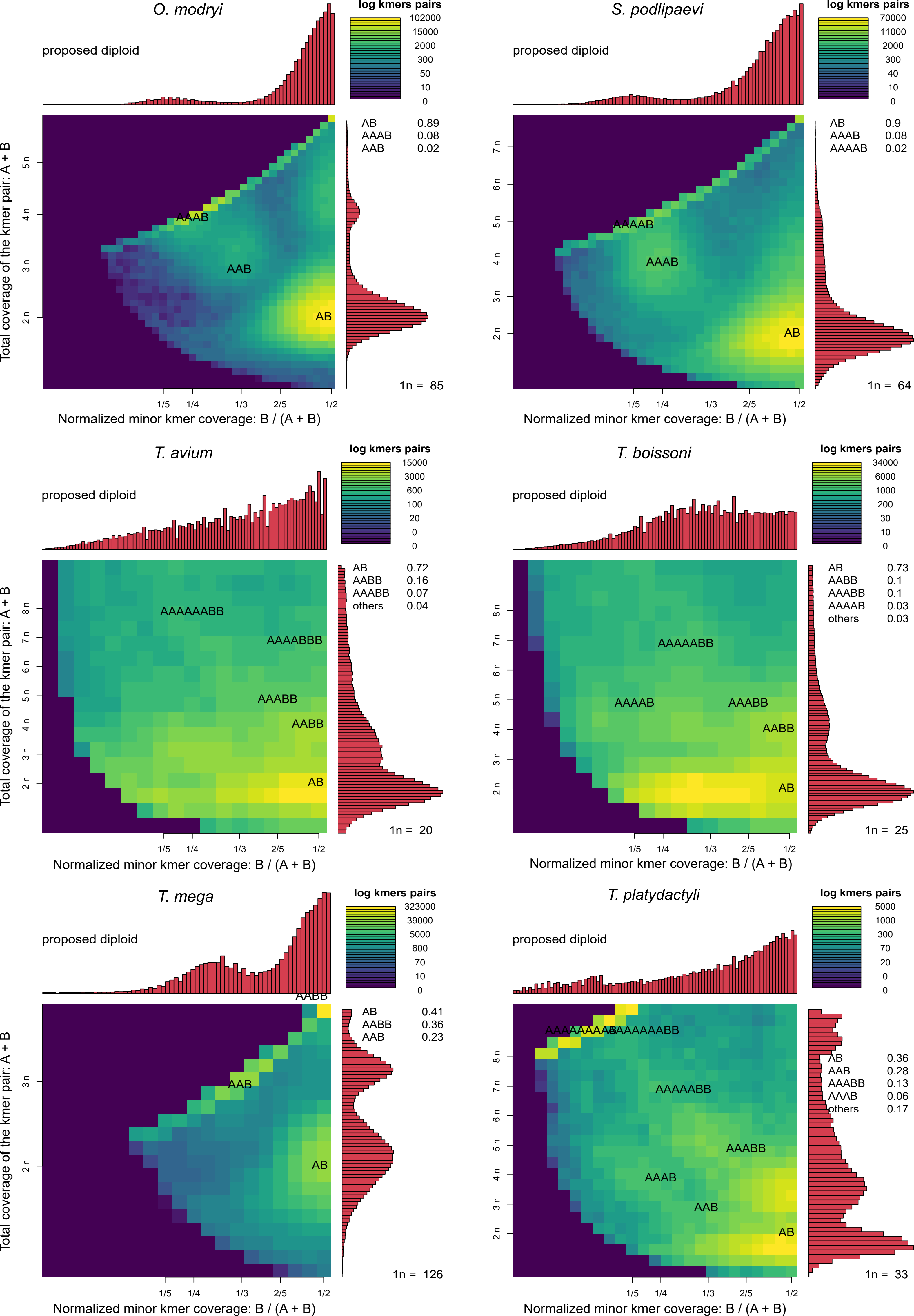
O. modryi
proposed diploid
log kmers pairs
102000
15000
2000
300
50
10
0
AB 0.89
AAAB 0.08
AAB 0.02
AAAB
AAB
AB
1n = 85
Total coverage of the kmer pair: A + B
Normalized minor kmer coverage: B / (A + B)
S. podlipaevi
proposed diploid
log kmers pairs
70000
11000
2000
300
40
10
0
AB 0.9
AAAB 0.08
AAAAB 0.02
AAAAB
AAAB
AB
1n = 64
Normalized minor kmer coverage: B / (A + B)
T. avium
proposed diploid
log kmers pairs
15000
3000
600
100
20
0
0
AB 0.72
AABB 0.16
AAABB 0.07
others 0.04
AAAAAABB
AAAABBB
AAABB
AABB
AB
1n = 20
Total coverage of the kmer pair: A + B
Normalized minor kmer coverage: B / (A + B)
T. boissoni
proposed diploid
log kmers pairs
34000
6000
1000
200
30
10
0
AB 0.73
AABB 0.1
AAABB 0.1
AAAAB 0.03
others 0.03
AAAAABB
AAAAB
AAABB
AABB
AB
1n = 25
Normalized minor kmer coverage: B / (A + B)
T. mega
proposed diploid
log kmers pairs
323000
39000
5000
600
70
10
0
AB 0.41
AABB 0.36
AAB 0.23
AABB
AAB
AB
1n = 126
Total coverage of the kmer pair: A + B
Normalized minor kmer coverage: B / (A + B)
T. platydactyli
proposed diploid
log kmers pairs
5000
1000
300
70
20
0
0
AB 0.36
AAB 0.28
AAABB 0.13
AAAB 0.06
others 0.17
AAAAABB
AAABB
AAAB
AAB
AB
1n = 33
Normalized minor kmer coverage: B / (A + B)

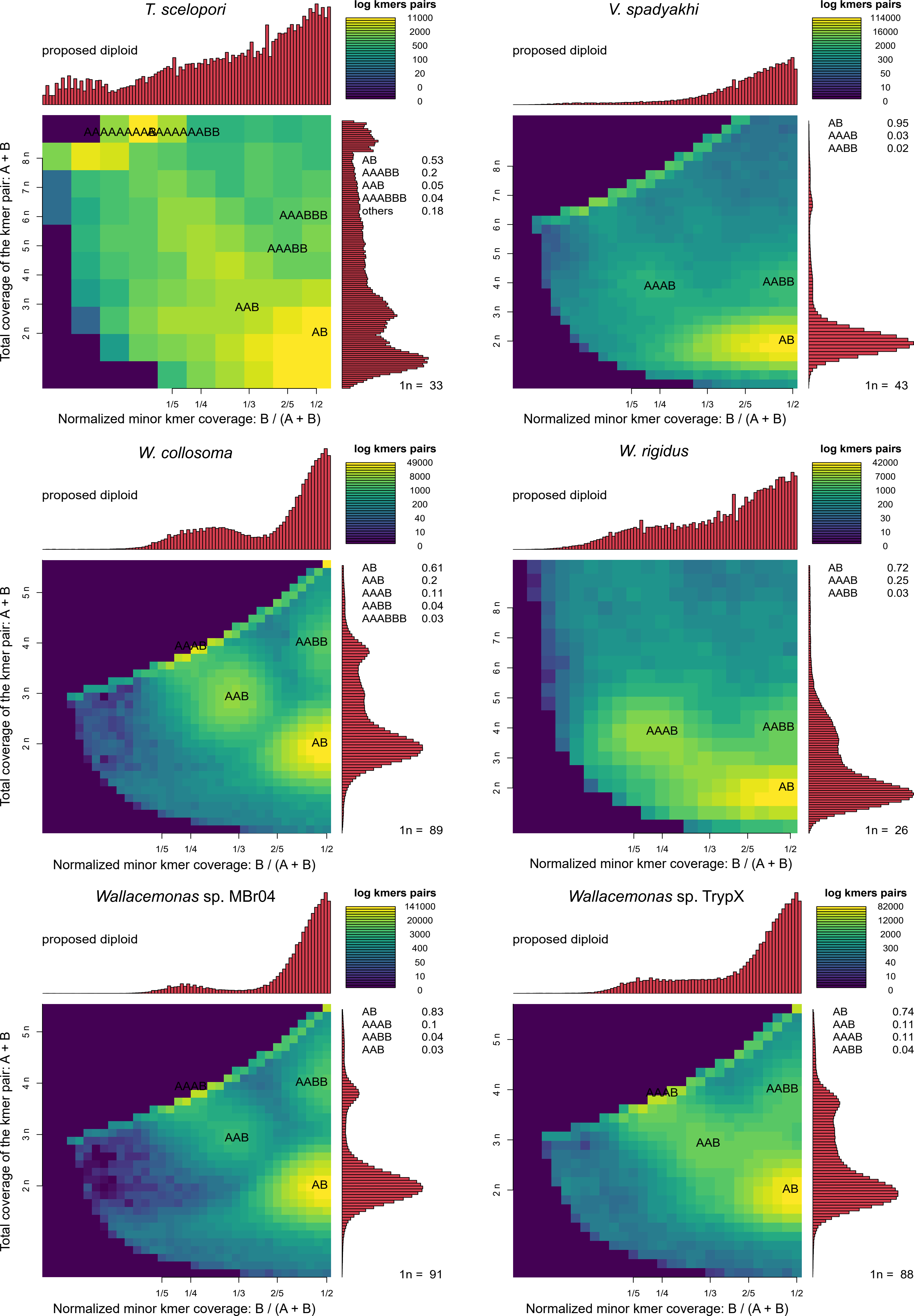
T. scelopori
proposed diploid
log kmers pairs
11000
2000
500
100
20
0
0
AB 0.53
AAABB 0.2
AAB 0.05
AAABBB 0.04
others 0.18
AAABBB
AAABB
AAB
AB
1n = 33
Total coverage of the kmer pair: A + B
2 n
3 n
4 n
5 n
6 n
7 n
8 n
1/5
1/4
1/3
2/5
1/2
Normalized minor kmer coverage: B / (A + B)
V. spadyakhi
proposed diploid
log kmers pairs
114000
16000
2000
300
50
10
0
AB 0.95
AAAB 0.03
AABB 0.02
AAAB
AABB
AB
1n = 43
2 n
3 n
4 n
5 n
6 n
7 n
8 n
1/5
1/4
1/3
2/5
1/2
Normalized minor kmer coverage: B / (A + B)
W. collosoma
proposed diploid
log kmers pairs
49000
8000
1000
200
40
10
0
AB 0.61
AAB 0.2
AAAB 0.11
AABB 0.04
AAABBB 0.03
AAAB
AABB
AAB
AB
1n = 89
Total coverage of the kmer pair: A + B
2 n
3 n
4 n
5 n
1/5
1/4
1/3
2/5
1/2
Normalized minor kmer coverage: B / (A + B)
W. rigidus
proposed diploid
log kmers pairs
42000
7000
1000
200
30
10
0
AB 0.72
AAAB 0.25
AABB 0.03
AAAB
AABB
AB
1n = 26
2 n
3 n
4 n
5 n
6 n
7 n
8 n
1/5
1/4
1/3
2/5
1/2
Normalized minor kmer coverage: B / (A + B)
Wallacemonas sp. MBr04
proposed diploid
log kmers pairs
141000
20000
3000
400
50
10
0
AB 0.83
AAAB 0.1
AABB 0.04
AAB 0.03
AAAB
AABB
AAB
AB
1n = 91
Total coverage of the kmer pair: A + B
2 n
3 n
4 n
5 n
1/5
1/4
1/3
2/5
1/2
Normalized minor kmer coverage: B / (A + B)
Wallacemonas sp. TrypX
proposed diploid
log kmers pairs
82000
12000
2000
300
40
10
0
AB 0.74
AAB 0.11
AAAB 0.11
AABB 0.04
AAAB
AABB
AAB
AB
1n = 88
2 n
3 n
4 n
5 n
1/5
1/4
1/3
2/5
1/2
Normalized minor kmer coverage: B / (A + B)

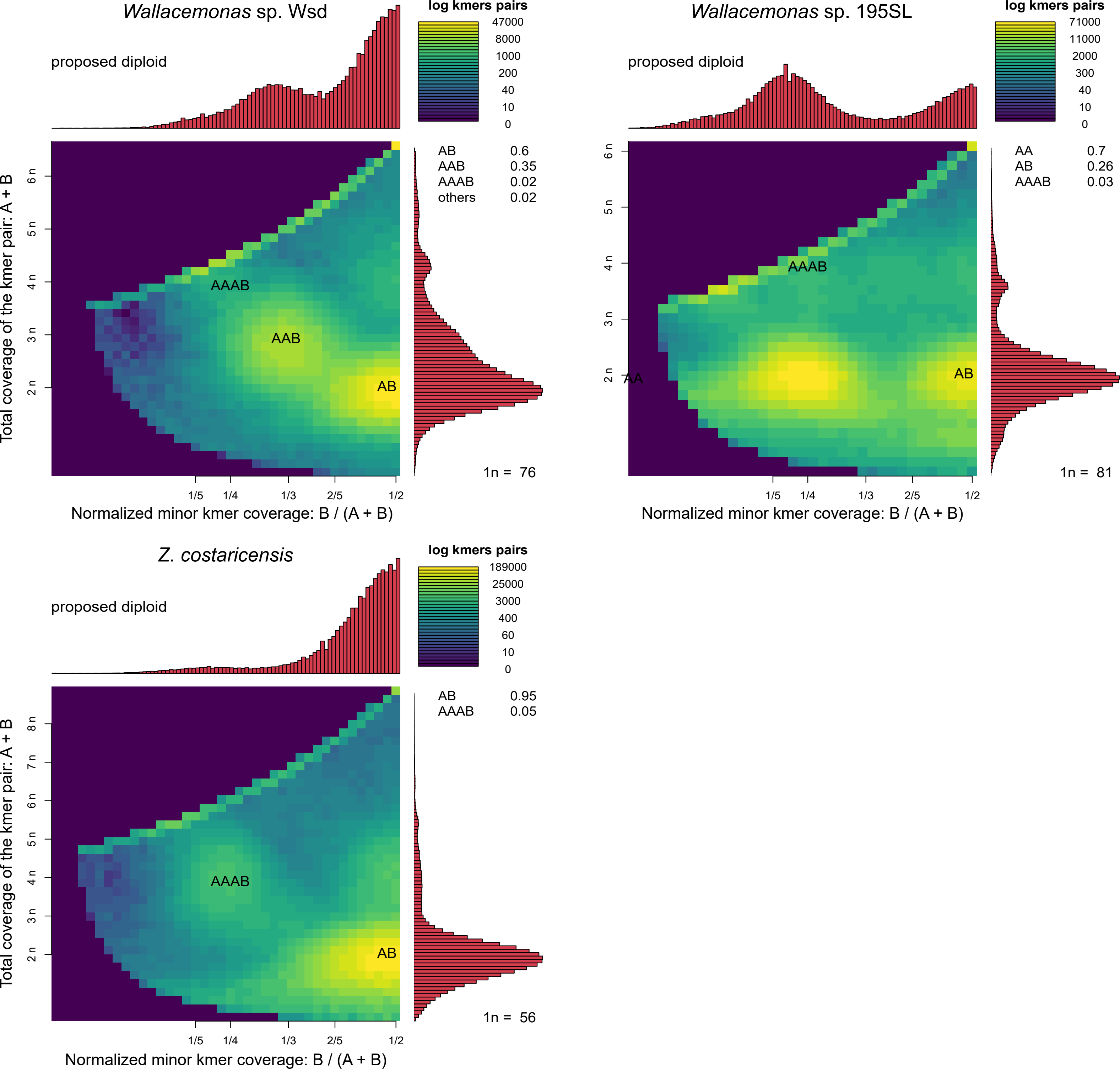

Wallacemonas sp. Wsd
proposed diploid
log kmers pairs
47000
8000
1000
200
40
10
0
AB 0.6
AAB 0.35
AAAB 0.02
others 0.02
AAAB
AAB
AB
1n = 76
6 n
5 n
4 n
3 n
2 n
Total coverage of the kmer pair: A + B
1/5
1/4
1/3
2/5
1/2
Normalized minor kmer coverage: B / (A + B)
Wallacemonas sp. 195SL
proposed diploid
log kmers pairs
71000
11000
2000
300
40
10
0
AA 0.7
AB 0.26
AAAB 0.03
AAAB
AA
AB
1n = 81
6 n
5 n
4 n
3 n
2 n
1/5
1/4
1/3
2/5
1/2
Normalized minor kmer coverage: B / (A + B)
Z. costaricensis
proposed diploid
log kmers pairs
189000
25000
3000
400
60
10
0
AB 0.95
AAAB 0.05
AAAB
AB
1n = 56
8 n
7 n
6 n
5 n
4 n
3 n
2 n
Total coverage of the kmer pair: A + B
1/5
1/4
1/3
2/5
1/2
Normalized minor kmer coverage: B / (A + B)
