# Supplementary material for: Shining the spotlight on the neglected: new high-quality genome assemblies as a gateway to understanding the evolution of Trypanosomatidae
Source: BMC Genomics. 2023 Aug 21;24:471. doi: 10.1186/s12864-023-09591-z (PMC10441713; doi:10.1186/s12864-023-09591-z)
Supplement: Supplementary file 8 — Additional file 8. Examples of command lines used for the genome assembly and downstream analyses. [file 12864_2023_9591_MOESM8_ESM.docx]

**Additional file 8.** Examples of command lines used for the genome assembly and downstream analyses.

1. Read trimming using Fastp v.0.20.1

| fastp --detect_adapter_for_pe --trim_poly_g --overrepresentation_analysis --cut_front --cut_front_window_size 1 --cut_front_mean_quality=20 --cut_tail --cut_tail_window_size 1 --cut_tail_mean_quality=20 --average_qual=30 --length_required 75 -i R1.fastq.gz -I R2.fastq.gz -o R1_trimmed-l75.fastq -O R2_trimmed-l75.fastq --thread 16 |
| --- |

1. Multiple sequence alignment-based error correction using Karect

| threads='80'  R1='R1_trimmed-l75.fastq'  R2='R2_trimmed-l75.fastq'  asm='asm.fasta'  karect -correct -threads=$threads -matchtype=hamming -celltype=diploid -inputfile=$R1 -inputfile=$R2  karect -align -threads=$threads -matchtype=hamming -inputfile=$R1 -inputfile=$R2 -refgenomefile=$asm -alignfile=align.txt  karect -eval -threads=$threads -matchtype=hamming -inputfile=$R1 -inputfile=$R2 -resultfile='karect_'$R1 -resultfile='karect_'$R2 -refgenomefile=$asm -alignfile=align.txt -evalfile=eval.txt |
| --- |

1. Genome assembly using SPAdes v.3.13.0

| spades.py --pe1-1 R1_trimmed-l75.fastq --pe1-2 R2_trimmed-l75.fastq -t 40 -o . |
| --- |

1. Genome assembly and scaffolding using Platanus v.1.2.4

| platanus assemble -o platanus_asm -f R?_trimmed-l75.fastq -t 40  platanus scaffold -o platanus_scaf_rnd1 -c contig.fa -IP1 R?_trimmed-l75.fastq |
| --- |

1. Gap-filling with GapCloser v.1.12

| GapCloser -b config.file -a asm_scaffold.l500.fasta -o asm_scaffold.l500.fasta_gapCloser_rnd1.fasta -t 20 |
| --- |

1. Read mapping

| R1='R1_trimmed-l75.fastq'  R2='R2_trimmed-l75.fastq'  asm='asm_scaffold.l500.fasta'  base='base_name'  bwa-mem2 index -p $base'_bwa2.index' $asm \| tee index.log    bwa-mem2 mem -M $base'_bwa2.index' $R1 $R2 -o $base'_bwa2.sam' -t 20 \| tee alignment.log  samtools faidx $asm 2>&1 \| tee samFai.log  samtools view --threads 10 -S -b -u -t $asm'.fai' $base'_bwa2.sam' -o $base'_bwa2.bam' 2>&1 \| tee samView.log  samtools sort $base'_bwa2.bam' -o $base'_bwa2.sorted.bam' --threads 40 \| tee samtools_sort.log  samtools index $base'_bwa2.sorted.bam' -@ 40 \| tee log samtools_index.log  bamtools stats -insert -in $base'_bwa2.sorted.bam' > stats_bamtools.tsv |
| --- |

1. Assembly filtering and decontamination

| asm='asm_scaffold.l500.fasta'  blastn_out='blastn.tsv'  bam='asm_DNA_bwa2.sorted.bam'  blastn -query $asm -max_target_seqs 10 -max_hsps 1 -evalue 1e-20 -db nt -outfmt '6 qseqid staxids bitscore std stitle sscinames qcovs' -num_threads 80 -out $blastn_out  cut -f1-14 $blastn_out > blastn-TO-BLOB.tsv  blobtools create -i $asm -b $bam -t blastn-TO-BLOB.tsv  blobtools view -i blobDB.json  blobtools plot -i blobDB.json --format 'pdf' |
| --- |

1. Repeat identification and soft-masking

| db='db_repeatModeler'  asm='asm.fasta'  #RepeatModeler  screen -L -Logfile RunDFAMTools.log bash dfam-tetools.sh  BuildDatabase -name $db $asm \| tee BuildDatabase.log  RepeatModeler -LTRStruct -database $db -pa 20 \| tee RepeatModeler.log  #RepeatMasker  RepeatMasker -lib ../repeatModeler/families.fa -xsmall -gff -pa 20 $asm -dir . |
| --- |

1. Duplicates removal and variant calling

| deDup_bam='duplicatesRemoved.bam'  asm='asm_softMasked.fasta'  #duplicates removal  gatk MarkDuplicates -I asm_DNA_bwa2.sorted.bam -O $deDup_bam -M duplicatesRemoved_metrics.txt --REMOVE_DUPLICATES true --MAX_RECORDS_IN_RAM 100000000 --MAX_FILE_HANDLES_FOR_READ_ENDS_MAP 1000  samtools index $deDup_bam -@ 10  samtools faidx $asm  #variants calling  platypus callVariants --bamFiles=$deDup_bam --refFile=$asm --output=only_indels.vcf --minReads 3 --genSNPs 0 --genIndels 1 --nCPU 40  gatk CreateSequenceDictionary -R $asm -O asm_softMasked.dict  gatk AddOrReplaceReadGroups -I $deDup_bam -O duplicatesRemoved_w_read_groups.bam -RGID 4 -RGLB lib1 -RGPL illumina -RGPU unit1 -RGSM 20  samtools index duplicatesRemoved_w_read_groups.bam -@ 10  gatk SortVcf -I only_indels.vcf -O only_indels.sorted.vcf --SEQUENCE_DICTIONARY asm_softMasked.dict  java -jar GenomeAnalysisTK_3.8/GenomeAnalysisTK.jar -T RealignerTargetCreator -R $asm -I duplicatesRemoved_w_read_groups.bam --known only_indels.sorted.vcf -o forIndelRealigner.intervals -nt 25  java -jar GenomeAnalysisTK_3.8/GenomeAnalysisTK.jar -T IndelRealigner -R $asm -I duplicatesRemoved_w_read_groups.bam -known only_indels.sorted.vcf --targetIntervals forIndelRealigner.intervals -o duplicatesRemoved_w_read_groups_indelRealigner.bam  platypus callVariants --genIndels=0 --bamFiles=duplicatesRemoved_w_read_groups_indelRealigner.bam --refFile=$asm --output=MarkedDuplicates_w_read_groups_realigned.vcf --nCPU 40  #vcf validation  gatk ValidateVariants --variant MarkedDuplicates_w_read_groups_realigned.vcf --reference $asm 2> validate.vcf.report.tsv  #SNPs assessment  gatk VariantsToTable -V MarkedDuplicates_w_read_groups_realigned.vcf -F CHROM -F POS -F TYPE -F TRANSITION -F HET -F HOM-REF -F HOM-VAR -F VAR -F MULTI-ALLELIC -GF AD -O variants.table.tsv  #Count total and homozygous (column 7 “HOM-VAR”) SNPs  awk '$3 == "SNP" { count++ } END { print "Total SNPs:\t"count } ($3 == "SNP") && ($7 == 1) {count1++} END {print "Homozygous SNPs:\t"count1} END {print "Homozygous percentage:\t"(count1*100)/count}' variants.table.tsv > SNPs_count.tsv |
| --- |

1. Calculation of coverage mean and median for ploidy analysis

| fasta='asm_softMasked.fasta'  bam='duplicatesRemoved_w_read_groups_indelRealigner.bam'  bed='*.regions.bed'  base_name='name'  #get 50 longest scaffolds, their length  export LC_ALL=C  seqkit fx2tab --length --name --header-line $fasta \| sort -g -r -k2 \| head -50 \|  awk '{ len=length($2); res=""; for (i=0;i<=len;i++) { res=substr($2,len-i+1,1) res;  if (i > 0 && i < len && i % 3 == 0) { res = "," res } }; print $1"\t"res"" }' \|  sed 's/_lengt.*\t/\t/g' \| sed '1i\scaff\tlength' > scaffolds_length.tsv  # Calculate mean scaffold coverage  mosdepth $base_name $bam --fast-mode --by 1000 --no-per-base -t 40  gunzip *.regions.bed.gz  cat $bed \| datamash --full median 4 \| cut -f1,5  cat $bed \| datamash --full mean 4 \| cut -f1,5  # Calculate median of genome coverage  cut -f1 scaffolds_length.tsv \| sed -e 's/^/grep "/g' -e 's/$/" *.regions.bed \| datamash --full median 4 \| cut -f1,5/g' > get_median_mean_50_largest_scaf.sh ;  sh get_median_mean_50_largest_scaf.sh \| sed 's/_length.*\t/\t/g' \| sed '1i\scaff\tmean_cov' > median_coverage_scaffolds.tsv  paste scaffolds_length.tsv median_coverage_scaffolds.tsv > scaffolds_length_cov.tsv |
| --- |

1. KAT hist - histogram with the number of distinct k-mers from the reads having a given frequency

| kat hist -m 27 -t 10 -o kat-hist_m27.hist ../../karect/karect_R?_trimmed-l75.fastq \| tee hist_dft_m27.log |
| --- |

1. KAT comp - evaluation of assembly completeness and artifacts

| kat comp -t 10 -m 27 -o kat_comp_m27 -p pdf 'karect_R1_trimmed-l75.fastq' asm.fasta \| tee kat_comp_m27.log |
| --- |

1. Ploidy estimation using Smudgeplot

| #The input is a set of trimmed genome sequencing reads  #1st - Give KMC all the files with trimmed reads to calculate k-mer frequencies and then generate a histogram of k-mers:  mkdir tmp  ls ../karect/*.fastq.gz > FILES  kmc -k27 -t16 -m64 -ci1 @FILES kmcdb tmp \| tee kmc.log  kmc_tools transform kmcdb histogram kmcdb_k27.hist  #2nd - kmer extraction  L=$(smudgeplot.py cutoff kmcdb_k27.hist L)  U=$(smudgeplot.py cutoff kmcdb_k27.hist U)  #3rd - extract kmers in the coverage range from L to U using kmc_tools.  kmc_tools transform kmcdb -ci"$L" -cx"$U" reduce kmcdb_L"$L"_U"$U"  #4th - run smudge_pairs on the reduced file to compute the set of kmer pairs.  smudge_pairs kmcdb_L"$L"_U"$U" kmcdb_L"$L"_U"$U"_coverages.tsv kmcdb_L"$L"_U"$U"_pairs.tsv > kmcdb_L"$L"_U"$U"_familysizes.tsv  #5th - generate the smudgeplot using the coverages of the identified kmer pairs  smudgeplot.py plot kmcdb_L"$L"_U"$U"_coverages.tsv |
| --- |
